# Synthesis of Novel Pyrido[4,3-*e*][1,2,4]triazino[3,2-*c*][1,2,4]thiadiazine 6,6-dioxide Derivatives with Potential Anticancer Activity

**DOI:** 10.3390/molecules21010041

**Published:** 2015-12-29

**Authors:** Jarosław Sławiński, Aleksandra Grzonek, Beata Żołnowska, Anna Kawiak

**Affiliations:** 1 Department of Organic Chemistry, Medical University of Gdańsk, Al. Gen. J. Hallera 107, 80-416 Gdańsk, Poland; aleksandra@gumed.edu.pl (A.G.); zolnowska@gumed.edu.pl (B.Ż.); 2 Department of Biotechnology, Intercollegiate Faculty of Biotechnology, University of Gdańsk and Medical University of Gdańsk, Ul. Kładki 24, 80-822 Gdańsk, Poland; kawiak@biotech.ug.edu.pl; 3 Department of Human Physiology, Medical University of Gdańsk, Ul. Tuwima 15, 80-210 Gdańsk, Poland

**Keywords:** pyrido[4,3-*e*][1,2,4]triazino[3,2-*c*][1,2,4]thiadiazine, 6,6-dioxides, synthesis, NMR studies, cytotoxic activity

## Abstract

A series of novel 3-/2,3-substituted pyrido[4,3-*e*][1,2,4]triazino[3,2-*c*][1,2,4]thiadiazine 6,6-dioxides **4**–**28** have been synthesized by the reaction of 3-amino-2-(4-thioxo-1,4-dihydropyridin-3-yl-sulfonyl)guanidine with either 2-oxoalkanoic acids and its esters, or phenylglyoxylic hydrates in glacial acetic acid. Some of them exhibited reasonable or moderate anticancer activity toward human cancer cell lines, HCT-116, MCF-7 and HeLa. The structure of this novel heterocyclic ring system was confirmed by ^1^D-NMR and ^2^D-NMR spectroscopic data including COSY, ROESY and HMBC, elemental analyses and MS spectrometry.

## 1. Introduction

**Figure 1:**
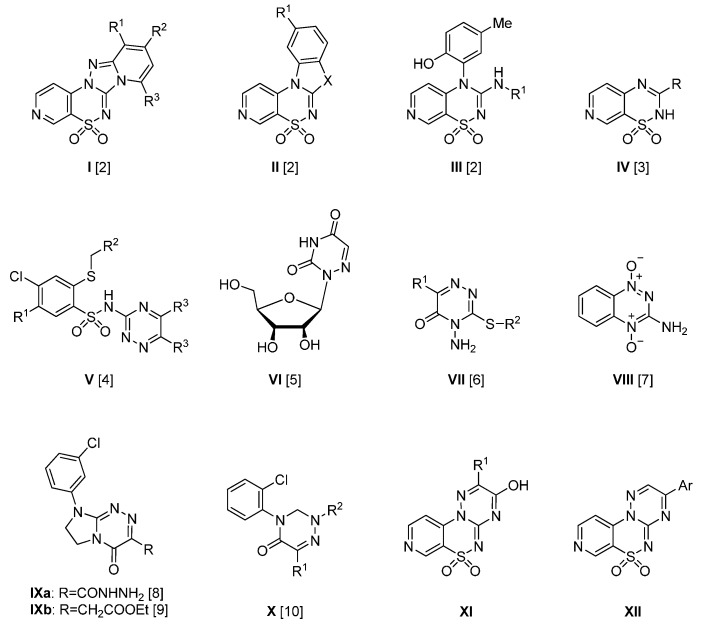
Structures of known anticancer and antibacterial pyridinethiadiazine derivatives **I**–**III** [2], anticancer and tuberculostatic 3-heteroaryl-2*H*-pyrido[4,3-*e*][1,2,4]thiadiazines **IV** [3], anticancer 1,2,4-triazines **V**–**X** [4,5,6,7,8,9,10] and novel 3-hydroxypyrido[4,3-*e*][1,2,4]triazino[3,2-*c*][1,2,4]thiadiazine 6,6-dioxides **XI** as well as 3-aryl-pyrido[4,3-*e*][1,2,4]triazino [3,2-*c*][1,2,4]thiadiazine 6,6-dioxides **XII**.

Cancer is the second most life threatening non-communicable disease after cardiovascular disease, according to the World Health Organization [1]. Despite the availability of numerous anticancer drugs, cancer is still hard to cure, especially without exhibiting any side effects. Due to the serious toxicity of conventional cytotoxic medicines contemporary medicinal chemistry is focused on the development of potent and selective anticancer drugs. Heterocyclic scaffolds play an important role for the design of novel drugs by enhancing their biological effects when fused with other ring systems. Among the many heteroaromatic rings, pyridothiadiazines constitutes an important framework of anticancer and antibacterial agents (**I**–**III**, Figure 1) [2] as well as anticancer and tuberculostatic compounds (**IV**, Figure 1) [3]. At the same time, there are also many reports indicating the significant anticancer properties of the 1,2,4-triazine fragment (**V**–**X**, Figure 1) [4,5,6,7,8,9,10]. It is known that 2-alkylthio-5-chloro-*N*-(1,2,4-triazin-yl)benzenesulfonamides **V** exhibit anticancer activity against colon, CNS, melanoma, ovarian, breast, renal, and leukemia cell lines [4]. The 6-azauridine **VI**, an inhibitor of *de novo* pyrimidine biosynthesis, has been described as an effective agent for inducing remission of acute myelocytic leukemia in children [5]. Other analogs of 6-azauridine, the *S*-alkyl derivatives of 1,2,4-triazinone **VII**, present cytotoxic activities against human breast cancer (MCF-7), colon carcinoma (HCT-116) and hepatocellular carcinoma (Hep-G2) cell lines [6]. Tirapazamine **VIII** is a known bioreductive hypoxia-selective cytotoxin, currently undergoing a variety of phase I, II and III clinical trials for the treatment of various human cancers, including non-small cell lung, cervical, head and neck, and ovarian cancers. Tirapazamine derives its medicinal activity by inducing DNA damage in poorly oxygenated tumor cells [7]. The heterobicyclic derivative bearing the 1,2,4-triazine moiety **IXa** have demonstrated remarkable inhibitory effects against uterus cancer (SiHa) cells and human colon adenocarcinoma (LS 180) cells while simultaneously inducing DNA cleavage [8]. Moreover, its close analog **IXb** was identified as capable of inducing significantly higher levels of necrotic cells in human breast cancer (T47D) cell lines and human cervical epithelial carcinoma (HeLa) cells [9]. Some of 1,2,4-triazine-5-ones **X** have distinct antiptoliferative activities against the chronic myeloid leukemia (K-562) cell line combined with a low cytotoxicity [10].

It has been known that arylsulfonamides exhibit a variety of biological activities including anticancer properties [11,12]. Our previous reports underlined the anticancer activity of arylsulfonamide analogues containing various heterocyclic ring systems attached to the benzenesulfonamide core [13,14,15,16,17,18]. Recently we have developed of a new method for the synthesis of 2,3-diaryl-9,9-dioxo-1*H*-9-thia-1,4,4a,7,10-pentaazaphenanthrene-2-ols [19]. This original class of compounds prompted us to investigate anticancer activity of arylsulfonamides modified by pyridothiadiazine and 1,2,4-triazine cores. In our research for novel various substituted pyrido[4,3-*e*][1,2,4]triazino-[3,2-*c*][1,2,4]thiadiazine 6,6-dioxides (**XI**,**XII**, Figure 1) with potential anticancer activity, we developed synthetic pathways for their preparation and evaluated their cytotoxic activity toward human cancer cell lines HCT-116, HeLa and MCF-7.

## 2. Results

### 2.1. Chemistry

**Scheme 1:**
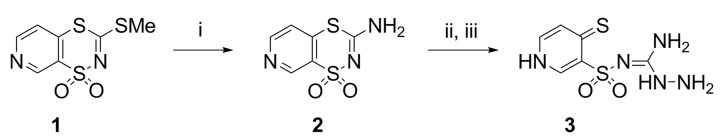
The three-step reaction for the synthesis of 3-amino-2-(4-thioxo-1,4-dihydropyridin-3-yl-sul-fonyl)guanidine (**3**). *Reagents, conditions and yields*: (i) 1 equiv. 25% NH_3_ aq, EtOH, 120 h, rt, 95%; (ii) 2 equiv. 99% N_2_H_4_ hydrate, dry MeOH, 30–40 h, 88%; (iii) AcOH, 88%.

The synthesis of the key starting material, *i.e.*, 3-amino-2-(4-thioxo-1,4-dihydropyridin-3-yl-sul-fonyl)guanidine (**3**) was achieved in a three-step reaction sequence starting from 3-methylthio-1,1-dioxopyrido[4,3-*e*]-1,4,2-dithiazine (**1**) according to the previously described procedure [19] (Scheme 1).

**Scheme 2:**
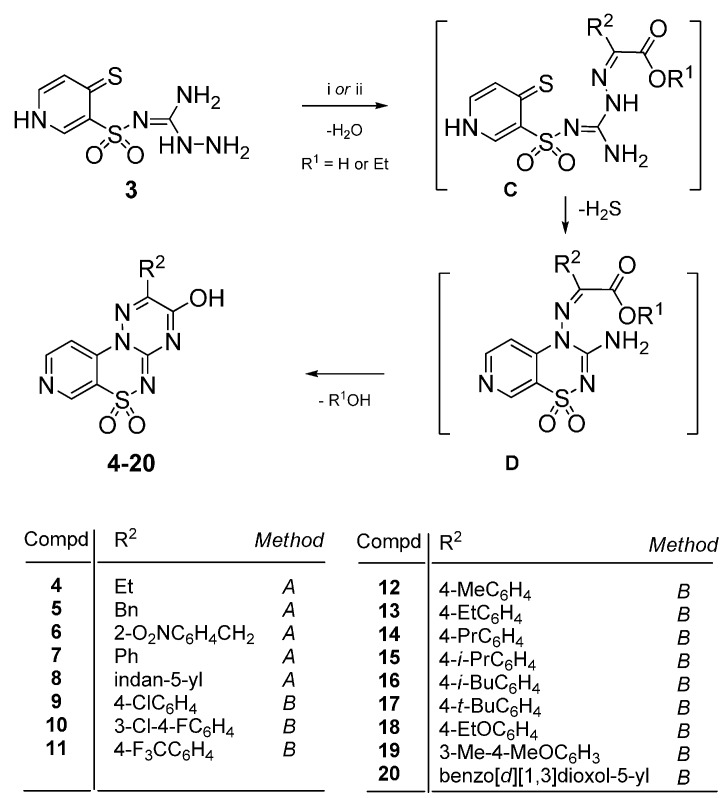
Proposed mechanism for the formation of 2-*R*^2^-3-hydroxypyrido[4,3-*e*][1,2,4]triazino[3,2-*c*][1,2,4]thiadiazine 6,6-dioxides **4**–**20**. *Reagents and conditions*: *Method A*: (i) the appropriate 2-oxo-alkanoic acid, (1.0 equiv.), glacial acetic acid, reflux 50–90 h; *Method B*: (ii) the appropriate ethyl phenylglyoxylate (1.0 equiv.), glacial acetic acid, reflux 22–26 h.

The desired 2-*R*^2^-3-hydroxypyrido[4,3-*e*][1,2,4]triazino[3,2-*c*][1,2,4]thiadiazine 6,6-dioxides **4**–**8** and **9**–**20** were obtained in facile “one-pot” reactions by treatment of aminoguanidine **3** with the appropriate 2-oxoalkanoic acids in glacial acetic acid at reflux for 50–90 h (compds. **4**–**8**; *Method A*). Optimization of the reaction conditions by use of an equimolar amount of the appropriate esters in boiling glacial acetic acid *i.e.*, ethyl phenylglyoxylate, caused a significant shortening of the reaction time up to 22–26 h (compds. **9**–**20**; *Method B*), as outlined in Scheme 2. The proposed mechanism of this reaction involves the initial formation of an intermediate condensation product of type **C**, which undergoes an intramolecular addition-elimination reaction (S_N_Ar) at the 4-position of the pyridine ring leading to the formation of pyridothiadiazine intermediate **D** with simultaneous H_2_S elimination. Final condensation reaction between carbonyl and amine group of **D** leads to the six-membered ring closure affording a novel heterocyclic ring system, namely pyrido[4,3-*e*][1,2,4]triazino[3,2-*c*][1,2,4]thiadiazine 6,6-dioxide (Scheme 2). Apparently, the elimination of hydrogen sulphide may be responsible for the elongation of the overall reaction time, due to the fact that gas H_2_S appears 10–15 h after the reaction initiation.

An attempt was made to apply 3-amino-2-(4-thioxo-1,4-dihydropyridin-3-ylsulfonyl)guanidine (**3**) to the reaction with phenylglyoxal hydrates due to the well-known fact that in acidic conditions the electrophlicity of its formyl group is superior to that of a carbonyl group in the reaction with substituted aminoguanidines [20]. Such an approach may open a convenient way to obtain the similar pyrido[4,3-*e*][1,2,4]triazino[3,2-*c*][1,2,4]thiadiazine 6,6-dioxide heterocyclic ring system possessing however, a phenyl moiety in the 3 position, and not in the 2 position as we previously proved in the reactions with 2-oxoalkanoic acids or esters (Scheme 2).

**Scheme 3:**
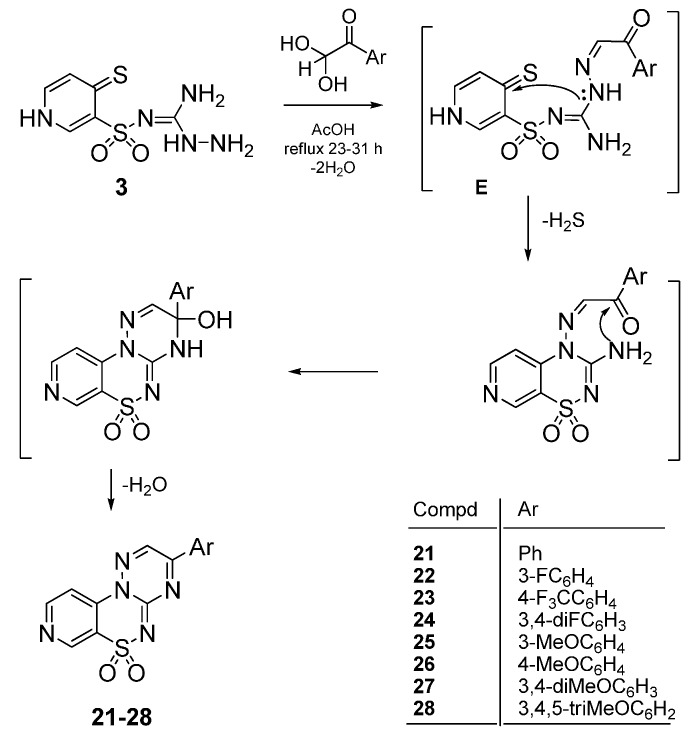
Proposed mechanism for the formation of 3-phenylpyrido[4,3-*e*][1,2,4]triazino[3,2-*c*][1,2,4]thiadiazine 6,6-dioxides **21**–**28**. *Reagents and conditions*: The appropriate phenylglyoxal hydrate (1.0 equiv.), glacial acetic acid, reflux 23–31 h.

Thus, treatment of aminoguanidine **3** with the appropriate phenylglyoxal hydrate in glacial acetic acid at reflux led to the expected 3-phenylpyrido[4,3-*e*][1,2,4]triazino[3,2-*c*][1,2,4]thiadiazine 6,6-dioxide derivatives **21**–**28**, as outlined in Scheme 3. We propose a similar mechanism for the formation of compounds **21**–**28** which consists in the initial formation of an intermediate condensation product of type **E** as a result of the regioselective nucleophilic attack of amine group on the 2,2-dihydroxyethanone carbon atom (Scheme 3).

The structures of **4**–**28** were confirmed by IR, NMR and MS data and elemental analyses. In the IR spectra of compounds **4**–**20** the characteristic OH group appeared as overlapping broad absorption bands in the 3543–3417 cm^−1^. In turn, in the ^1^H-NMR spectra of the compounds **4**–**28** the chemical shifts of protons H-7, H-9 and H-10 attributable to pyridine ring of the tricyclic fused ring system were found in the region of 9.06–9.25, 9.03–9.80 and 7.79–8.18 ppm, respectively (Figures S1–S28, see Supplementary Materials).

### 2.2. Anticancer Activity

Compounds **4**–**28** were evaluated *in vitro* for their effects on the viability of three human cancer cell lines: HCT-116 (colon cancer), HeLa (cervical cancer) and MCF-7 (breast cancer). The concentration required for 50% inhibition of cell viability IC_50_ was calculated and compared with the reference drug cisplatin, and the results are given in Table 1. The compounds **5**, **15**, **17**, **21**–**23** and **25**–**28** exhibited either reasonable, moderate or weak anticancer activity against HCT-116 cell line, while five of them *i.e.*, **22**, **23**, **25**, **26** and **28** were also potent against MCF-7 and HeLa human cancer cell lines, whereas remaining compounds **4**, **6**–**14**, **16**, **18**–**20** and **24** were essentially inactive in tested concentration range.

**Table 1:** Cytotoxicity of compounds **4**–**28** toward human cancer cell lines *.

Compounds	IC_50_ (μM) ^a^		
	HCT-116	HeLa	MCF-7
**4**	140 ± 7	370 ± 11	*
**5**	80 ± 2	220 ± 7	*
**6**	160 ± 2	700 ± 14	300 ± 12
**7**	250 ± 15	470 ± 9	*
**8**	240 ± 9	350 ± 7	320 ± 6
**9**	160 ± 3	*	650 ± 7
**10**	*	*	*
**11**	260 ± 15	*	*
**12**	180 ± 5	*	350 ± 21
**13**	110 ± 2	*	520 ± 15
**14**	*	*	*
**15**	84 ± 2	*	*
**16**	200 ± 4	*	*
**17**	90 ± 4	*	*
**18**	270 ± 5	310 ± 6	*
**19**	*	*	*
**20**	250 ± 20	*	550 ± 16
**21**	59 ± 1	190 ± 6	94 ± 2
**22**	13 ± 0.8	57 ± 2	25 ± 0.7
**23**	9 ± 0.2	25 ± 0.5	25 ± 1
**24**	170 ± 11	260 ± 8	240 ± 5
**25**	14 ± 1	56 ± 2	31 ± 1
**26**	42 ± 1	83 ± 2	97 ± 3
**27**	47 ± 1	110 ± 3	105 ± 3
**28**	26 ± 0.5	49 ± 1	35 ± 3
Cisplatin	3.8 ± 0.2	2.2 ± 0.2	3.0 ± 0.1

^a^ Analysis was performed using the MTT assay after 72 h of incubation. Values are expressed as the mean ± SD of at least three independent experiments. * Viability of cell lines at 100 μM of tested compounds were approximately 100%.

## 3. Discussion

### 3.1. Anticancer Activity

As shown in Table 1 the HCT-116 cell line exhibited the relatively highest susceptibility to compounds **5**, **15**, **17**, **21**–**23** and **25**–**28** with the average value of IC_50_ = 46.4 μM. Apparently, the presence of a mono- or tri-substituted phenyl moiety (Ar) at position 3 leads to the compounds with good profile of anticancer activity. Thus, the compounds **22**, **23**, **25**, **26** and **28** exhibited inhibition of growth toward not only HCT-116 cell line at IC_50_ = 9–42 μM, but also for the HeLa and MCF-7 cells in the concentration range of IC_50_ = 25–83 μM, and IC_50_ = 25–97 μM, respectively. Among them, compounds **22**, **23**, **25** and **28** having variously substituted phenyl ring at position 3, *i.e.*, **22** (Ar = 3-FC_6_H_4_), **23** (Ar = 4-F_3_CC_6_H_4_), **25** (Ar = 3-MeOC_6_H_4_) and **28** (Ar = 3,4,5-tri-MeOC_6_H_2_) exhibited relevant influence on cytotoxic activity toward HCT-116 cell line (IC_50_ = 9–26 μM).

Otherwise, the presence of unsubstituted phenyl ring **21** (Ar = Ph) or 3,4-disubstituted phenyl moieties **24** (Ar = 3,4-diFC_6_H_4_) and **27** (Ar = 3,4-diMeOC_6_H_3_) significantly decreases this activity. In general, the HeLa cell line was less susceptible to the tested compounds, with the exception of **23** (Ar = 4-F_3_CC_6_H_4_), which exhibited cytotoxic activity in the low micromolar range (IC_50_ = 25 μM), whereas **22** (Ar = 3-FC_6_H_4_), **25** (Ar = 3-MeOC_6_H_4_), **26** (Ar = 4-MeOC_6_H_4_) and **28** (Ar = 3,4,5-triMeOC_6_H_2_) inhibited cell growth in the higher concentration range IC_50_ = 49–83 μM. On the other hand, compounds **21** (Ar = Ph), **24** (Ar = 3,4-diFC_6_H_4_) and **27** (Ar = 3,4-diMeOC_6_H_3_) were inactive towards this cell line.

Cell growth inhibition of MCF-7 line occurred in the low concentration range IC_50_ = 25–35 μM by compounds **22** (Ar = 3-FC_6_H_4_), **23** (Ar = 4-F_3_CC_6_H_4_), **25** (Ar = 3-MeOC_6_H_4_) and **28** (Ar = 3,4,5-triMeOC_6_H_2_). Moreover, replacement of aryl substituent (Ar) in active compounds **25** and **28** by the differently substituted isomers, *i.e.*, **26** (Ar = 4-MeOC_6_H_2_) or **27** (Ar = 3,4-diMeOC_6_H_3_) caused significant decreases in activity of **26** (IC_50_ = 97 µM) or the loss of activity (**27**) in tested concentration range (Table 1).

We found, that compounds **22** (Ar = 3-FC_6_H_4_), **23** (Ar = 4-F_3_CC_6_H_4_), **25** (Ar = 3-MeOC_6_H_4_) and **28** (Ar = 3,4,5-triMeOC_6_H_2_) exhibited strong cytotoxic activity against all of the tested cell lines in the relatively low or moderate concentrations range IC_50_ = 9–57 μM.

To summarize the biological test results, the replacement of an aryl (Ar) substituent by a hydroxy (OH) functionality at position 3 with simultaneously shifting of the substituent *R^2^* = alkyl, benzyl, substituted aryl to the position 2 caused the loss of activity towards the HeLa and MCF-7 cell lines in compounds **7**–**20**. Among a series of 2,3-substituted derivatives only weak activity against HCT-116 cell lines for **5** (*R^2^* = Bn), **15** (*R^2^* = 4-*i*-PrC_6_H_4_) and **17** (*R^2^* = 4-*t*-BuC_6_H_4_) was observed.

**Figure 2:**
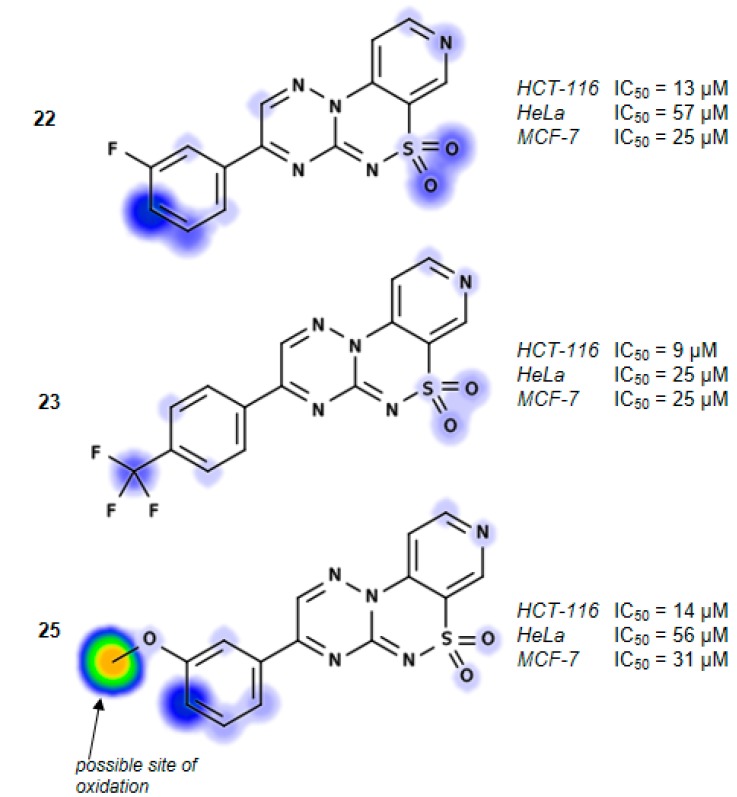

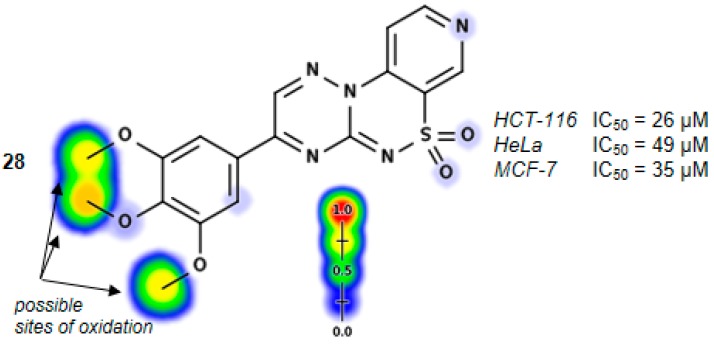
Sites of metabolism predicted for **22**, **23**, **25** and **28** by the XenoSite software [21]. The green color indicates more vulnerability to biotransformation than blue. Some significant differences are additionally pointed out.

The rational design of more effective and safe compounds may be supported by *in silico* approaches that predict the human *in vivo* metabolism and reactivity of small molecules. Therefore in the present studies we applied an on-line accessible tool for accurate prediction of xenobiotic metabolism sites, called XenoSite Cytochrome P450 Prediction Models [21] and estimated the stability of the most active compounds **22**, **23**, **25** and **28** in the presence of human liver microsomes. *In silico* results illustrated in Figure 2 show which atoms on a molecule are likely to be oxidized by human liver microsomes. Thus, it was found that the prominent compound **23** demonstrated not only the minimal IC_50_ values against tested cell lines, but also the high metabolic stability with regard to oxidative metabolic processes. In turn, drug toxicity, frequently described by the quantitative strength of a molecule’s reactivity with glutathione, was also predicted by use of the XenoSite Reactivity Model [22]. The results of the *in silico* analysis did not indicate any toxicity of compounds **22**, **23**, **25** and **28**.

### 3.2. NMR Studies

**Figure 3:**
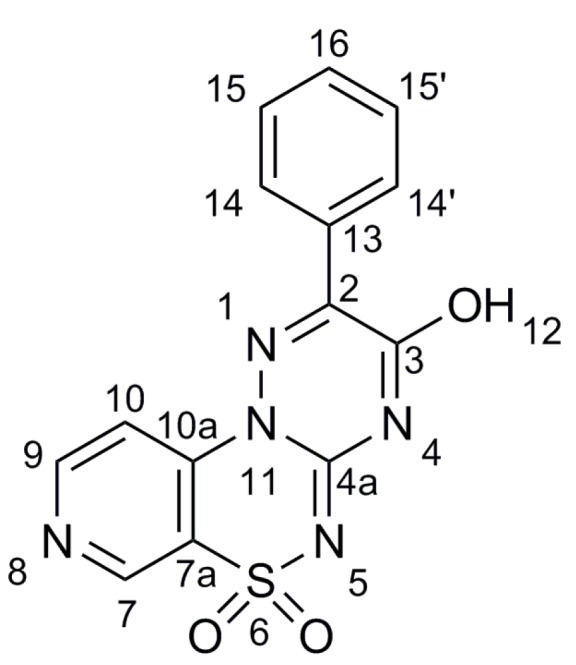
The structure of 2-phenyl-3-hydroxypyrido[4,3-*e*][1,2,4]triazino[3,2-*c*][1,2,4]thiadiazine 6,6-dioxide **7**; the atom numbers are correlated with the NMR results.

**Figure 4:**
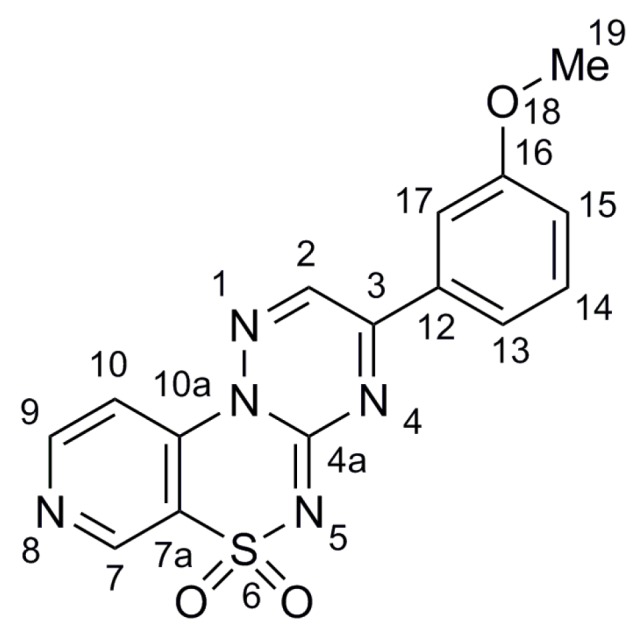
The structure of 3-(3-methoxyphenyl)pyrido[4,3-*e*][1,2,4]triazino[3,2-*c*][1,2,4]thiadiazine 6,6-dioxide **25**; the atom numbers are correlated with the NMR results.

The two dimensional NMR spectroscopy studies, consisting of COSY, ROESY and HMBC experiments, resulted in structure confirmation of two various substituted isomers such as 2-phenyl-3-hydroxypyrido[4,3-*e*][1,2,4]triazino[3,2-*c*][1,2,4]thiadiazine 6,6-dioxide **7** (Figure 3) and monosubstituted counterpart, *i.e.*, 3-(3-methoxyphenyl)pyrido[4,3-*e*][1,2,4]triazino[3,2-*c*][1,2,4]thiadiazine6,6-dioxide (**25**, Figure 4) by proton and carbon assignments, which are given in Table 2, Table 3, Table 4 and Table 5. The resonance signal of proton H7 in compound **7** was identified as the only one singlet showing correlations to four *sp*^2^ carbons C7a, C9, C10 and C10a in HMBC spectrum. The COSY spectrum of **7** allowed the connectivities within two structural blocks, namely C9-C10 and C14-C16 to be traced. The C7, C9-C10 and C14-C16 spin systems were distinguished by the presence of H7/H9 ROE and upon the C2/H14 heteronuclear correlation in HMBC spectrum. Finally, the assignments of quaternary *sp*^2^ carbon atoms C2, C7a, C10a and C13 were made upon several heteronuclear correlations observed in the HMBC spectrum, listed in Table 2 and Table 3.

**Table 2:** ^1^H-NMR data for **7**.

Proton No.	^1^H δ (ppm)	^3^*J* (HH) Contacts (Hz)	ROE Contacts
H-7	9.13	-	H-9
H-9	8.91	5.9 (H-10)	H-7
H-10	8.11	5.9 (H-9)	-
H-14, 14′	8.10	7.3 (H-15, 15′)	-
H-15, 15′	7.59	7.3 (H-14, 14′); 7.4 (H-16)	-
H-16	7.55	7.4 (H-15, 15′)	-

**Table 3:** ^13^C-NMR data for **7** with long-range C/H couplings observed in HMBC spectrum.

Carbon No.	^13^C δ (ppm)	HMBC Protons
C-2	147.5	H-14
C-3	154.3	-
C-4a	144.4	-
C-7	146.6	H-10
C-7a	120.8	H-7, H-9, H-10
C-9	154.4	H-7, H-10
C-10	111.6	H-7, H-9
C-10a	142.3	H-7, H-9, H-10
C-13	132.0	H-14
C-14, 14′	129.9	H-14, 14′, H-15, 15′
C-15, 15′	129.1	H-16
C-16	131.2	H-16

**Table 4:** ^1^H-NMR data for **25**.

Proton No.	^1^H δ (ppm)	^3^*J* (HH) Contacts (Hz)	ROE Contacts
H-2	9.52	-	H-17, H-13
H-7	9.23	-	-
H-9	9.02	5.8 (H-10)	-
H-10	8.16	5.8 (H-9)	-
H-13	8.03	7.9 (H-14)	H-2
H-14	7.62	7.9 (H-13), 7.9 (H-15)	-
H-15	7.39	~8.0 (H-14)	H-17, H-19
H-17	7.91	-	H-2, H-15, H-19
H-19	3.90	-	H-15, H-17

**Table 5:** ^13^C-NMR data for **25** with long-range C/H couplings observed in HMBC spectrum.

Carbon No.	^13^C δ (ppm)	HMBC Protons
C-2	146.8	-
C-3	161.9	H-2, H-13, H-17
C-4a	131.0	-
C-7	146.1	H-9
C-7a	120.3	H-7, H-9
C-9	153.8	H-7, H-10
C-10	111.5	H-7, H-9
C-10a	141.7	H-7, H-9, H-10
C-12	137.0	-
C-13	122.0	H-17, H-14, H-15
C-14	121.3	H-13, H-17, H-15
C-15	133.1	H-14
C-16	160.0	H-17, H-14, H-15
C-17	113.5	H-13, H-15
C-19	55.7	-

The resonance signals of protons H2, H7 and H17 in compound **25** were identified as singlets showing correlations to the appropriate *sp*^2^ carbon atoms, as follows: H2/C3, H7/C7a, H7/C9, H7/C10, H7/C10a, and H17/C3, H17/C13, H17/C16 in HMBC spectrum. The COSY spectrum showed ^1^H-^1^H correlations within two structural blocks, namely C9-C10 and C13-C15 to be traced. The C13-C15 spin system was distinguished by the presence of H2/H13, H15/H17 and H15/H19 ROEs and upon the C13/H14, C13/H15, C13/H17, C17/H13 and C17/H15 heteronuclear correlations. The presences of H2/H13, H2/H17 ROEs with C3/H2, C3/H13 and C3/H17 HBMC correlations revealed the position of the phenyl substituent. The assignments of quaternary *sp*^2^ carbons C3, C7a, C10a, and C16 were made upon several heteronuclear correlations observed in the HMBC spectrum, listed in Table 4 and Table 5.

## 4. Experimental Section

### 4.1. General Information

The melting points were determined on a SMP30 apparatus (Stuart, BIBY Scientific Ltd., Stone, UK) and are uncorrected. Infrared (IR) spectra were recorded on a Mattson Satellite FTIR spectrophotometer (Thermo, Madison, WI, USA). The NMR spectra were recorded on a Unity 500 Plus apparatus at 500 MHz (^1^H-NMR) and 125 MHz (^13^C-NMR) (Varian, Palo Alto, CA, USA). Chemical shifts are expressed as δ values in parts per million (ppm) relative to TMS as an internal standard, coupling constants (*J*) are given in Hertz; multiplicity in ^1^H-NMR is reported as singlet (s), broad singlet (br.s), doublet (d), triplet (t), and multiplet (m). The spectra of ^13^C- and one-dimensional ^1^H were collected with standard parameters. ROESY spectra was acquired in phase-sensitive mode with a spectral width of 3398 Hz. The ROESY spectrum was acquired with a mix time of 300 ms in a 1024 × 200 matrix with 24 accumulations per increment and processed in 1 K × 1 K matrix. COSY and HMBC experiments were performed with pulse field gradients. The COSY spectrum was acquired in 1024 × 256 matrix with 4 accumulations and processed in 1 K × 1 K matrix. The HMBC spectrum was acquired in phase sensitive mode. The spectral windows for ^1^H and ^13^C axes were 3398 Hz and 16341 Hz, respectively. Data were collected in 736 × 180 matrix and processed in 1 K × 1 K matrix. LC-MS analysis for compound **7**: LCMS-IT-TOF LC-20A mass spectrometer (Shimadzu Scientific Instruments, Columbia, MD, USA) equipped with an electrospray ionization source capillary voltage in positive ion mode +4.5 kV; Column: Jupiter 4 u Proteo 90 Å, 4.0 × 150 mm, 4 µm, Mobile Phase: A—grade water with 0.1% formic acid, B—0.1% formic acid in acetonitrile, linear gradient 50%–100% B in 45 min, Flow Rate: 0.2 mL/min. Elemental analyses for C, H and N were performed on a 2400 Series II CHN Elemental Analyzer (PerkinElmer, Shelton, CT, USA) and are in agreement with the theoretical values within ±0.4% range. Thin-layer chromatography (TLC) was performed on Kieselgel 60F_254_ plates (Merck, Darmstadt, Germany) and visualized by UV illumination. The commercially unavailable substrates were obtained according to the method described previously: 3-amino-1,1-dioxopyrido[4,3-*e*]-1,4,2-dithiazine (**2**) and 3-amino-2-(4-thioxo-1,4-dihydropyridine-3-yl-sulfonyl)guanidine (**3**) [19].

### 4.2. Synthesis

#### 4.2.1. General Procedure for the Preparation of 2-Substituted-3-hydroxypyrido[4,3-*e*][1,2,4]triazino[3,2-*c*][1,2,4]thiadiazine 6,6-dioxides **4**–**8**

*Method A:* The mixture of **3** (1 mmol) and the appropriate 2-oxoalkanoic acid (1 mmol) in glacial acetic acid (3 mL) was stirred at reflux for 50–90 h. After standing overnight at room the precipitate was filtered off and purified by extraction of contaminations with boiling ethanol. In this manner the following compounds **4**–**8** were obtained.

*2-Ethyl-3-hydroxypyrido[4,3-e][1,2,4]triazino[3,2-c][1,2,4]thiadiazine 6,6-dioxide* (**4**). The mixture of **3** and 2-oxobutanoic acid (0.102 g), gave compound **4** as a white solid powder (0.085 g, 30%) after 50 h of heating at reflux, m.p. 302–303 °C; IR (KBr) ν_max_ 3525 (OH), 1619 (C=C), 1586 (C=N), 1312, 1165 (SO_2_) cm^−1^; ^1^H-NMR (DMSO-*d*_6_) δ 1.22 (t, *J* = 7.32 Hz, 3H), 2.71 (q, *J* = 7.00 Hz, 2H), 7.98 (d, *J* = 5.86 Hz, 1H), 8.89 (d, *J* = 5,86 Hz, 1H), 9.08 (s, 1H) ppm; ^13^C-NMR (DMSO-*d*_6_): δ 10.11, 23.84, 111.23, 120.53, 142.33, 144.69, 146.52, 154.14, 154.23, 154.31 ppm; anal. C 42.69, H 3.07, N 24.70% calcd. for C_10_H_9_N_5_O_3_S, C 43.01, H 3.25, N 25.08%.

*2-Benyzl-3-hydroxypyrido[4,3-e][1,2,4]triazino[3,2-c][1,2,4]thiadiazine 6,6-dioxide* (**5**). The mixture of **3** and 2-oxo-3-phenylpropanoic acid (0.164 g), gave compound **5** as a white solid powder (0.076 g, 22%) after 90 h of heating at reflux, m.p. 303–305 °C; IR (KBr) ν_max_ 3425 (OH), 1638 (C=C), 1587 (C=N), 1321, 1178 (SO_2_) cm^−1^; ^1^H-NMR (DMSO-*d*_6_) δ 4.04 (br. s, 2H), 7.27 (m, 2H), 7.36 (m, 2H), 7.79 (br. s, 1H), 8.86 (br. s, 1H), 9.08 (br. s, 1H) ppm; ^13^C-NMR (DMSO-*d*_6_) δ 36.24, 111.02, 120.55, 127.53, 129.05, 130.04, 135.89, 142.29, 144.66, 146.59, 151.79, 154.15, 154.24 ppm; anal. C 52.45, H 3.07, N 20.12% calcd. for C_15_H_11_N_5_O_3_S, C 52.78, H 3.25, N 20.52%.

*3-Hydroxy-2-(2-nitrobenzyl)pyrido[4,3-e][1,2,4]triazino[3,2-c][1,2,4]thiadiazine 6,6-dioxide* (**6**). The mixture of **3** and 3-(2-nitrophenyl)-2-oxopropanoic acid (0.209 g) gave compound **6** as a yellow solid powder (0.119 g, 31%) after 90 h of heating at reflux, m.p. 277–279 °C; IR (KBr) ν_max_ 3442 (OH), 1636 (C=C), 1588 (C=N), 1311, 1177 (SO_2_) cm^−1^; ^1^H-NMR (DMSO-*d*_6_) δ 4.42 (s, 2H), 7.36 (d, *J* = 5.86 Hz, 1H), 7.64 (m, 1H), 7.75 (t, *J* = 7.32 Hz, 1H), 8.11 (d, *J* = 7.81 Hz, 1H), 8.80 (d, *J* = 5.86 Hz, 1H) 9.06 (s, 1H) ppm; ^13^C-NMR (DMSO-*d*_6_) δ 33.53, 110.43, 120.49, 125.57, 129.59, 130.39, 133.87, 134.42, 142.11, 144.54, 146.63, 149.64, 150.97, 154.11 ppm; anal. C 47.02, H 2.46, N 21.50% calcd. for C_15_H_10_N_6_O_5_S, C 46.63, H 2.61, N 21.75%.

*3-Hydroxy-2-phenylpyrido[4,3-e][1,2,4]triazino[3,2-c][1,2,4]thiadiazine 6,6-dioxide* (**7**). The mixture of **3** and 2-phenyl-2-oxoacetic acid (0.150 g), gave compound **7** as a white solid powder (0.150 g, 45%) after 60 h of heating at reflux, m.p. 349–350 °C; IR (KBr) ν_max_ 3424 (OH), 1629 (C=C), 1311, 1173 (SO_2_) cm^−1^; ^1^H-NMR (DMSO-*d*_6_) δ 7.55 (t, 1H), 7.59 (t, 2H), 8.10 (t, 2H), 8.11 (d, *J* = 5.86 Hz, 1H), 8.91 (d, *J* = 5.86 Hz, 1H), 9.13 (s, 1H) ppm; ^13^C-NMR (DMSO-*d*_6_) δ 111.56, 120.78, 129.14, 129.85, 131.23, 132.00, 142.29, 144.39, 146.60, 147.54, 154.26, 154.36 ppm; LC-MS IT-TOF *m*/*z* [M + H]^+^ calcd. for C_14_H_9_N_5_O_3_S: 327.32, found: 328.04, *t*_R_ = 22 min. Anal. C 50.99, H 3.07, N 21.05% calcd. for C_14_H_9_N_5_O_3_S, C 51.37, H 2.77, N 21.40%.

*2-(2,3-Dihydro-1H-inden-5-yl)-3-hydroxypyrido[4,3-e][1,2,4]triazino[3,2-c][1,2,4]thiadiazine 6,6-di-oxide* (**8**). The mixture of **3** and 2-(2,3-dihydro-1*H*-inden-5-yl)-2-oxoacetic acid (0.190 g), gave compound **8** as a white solid powder (0.098 g, 27%) after 50 h of heating at reflux, m.p. 349–350 °C; IR (KBr) ν_max_ 3425 (OH), 1637 (C=C), 1587 (C=N), 1320, 1186 (SO_2_) cm^−1^; ^1^H-NMR (DMSO-*d*_6_) δ 2.05 (br. s, 2H), 2.81–3.04 (m, 4H), 7.38 (d, *J* = 6.35 Hz, 1H), 7.89 (d, *J* = 6.35 Hz, 1H), 7.94 (br. s, 1H), 8.10 (br. s, 1H), 8.92 (br. s, 1H), 9.11 (br. s, 1H) ppm; ^13^C-NMR (DMSO-*d*_6_) δ 25.72, 32.93, 33.09, 111.57, 120.76, 124.91, 125.50, 128.14, 129.19, 142.30, 144.36, 144.74, 146.56, 147.70, 148.41, 154.34 ppm; anal. C 55.70, H 3.26, N 18.84% calcd. for C_17_H_13_N_5_O_3_S, C 55.58, H 3.57, N 19.06%.

#### 4.2.2. General Procedure for the Preparation of 2-Aryl-3-hydroxypyrido[4,3-*e*][1,2,4]triazino[3,2-*c*][1,2,4]thiadiazine 6,6-dioxides **9**–**20**

*Method B:* The mixture of **3** (1 mmol) and the appropriate ethyl phenylglyoxylate (1 mmol) in glacial acetic acid (3 mL) was stirred at reflux for 22–26 h. After standing overnight at room temperature the precipitate was filtered off and purified by extraction of the impurities with boiling ethanol. In this manner the following compounds **9**–**20** were obtained.

*2-(4-Chlorophenyl)-3-hydroxypyrido[4,3-e][1,2,4]triazino[3,2-c][1,2,4]thiadiazine 6,6-dioxide* (**9**). The mixture of **3** and ethyl 2-(4-chlorophenyl)-2-oxoacetate (0.213 g) gave compound **9** as a white solid powder (0.088 g, 24%), m.p. 366–368 °C; IR (KBr) ν_max_ 3431 (OH), 1634 (C=C), 1588 (C=N), 1323, 1180 (SO_2_) cm^−1^; ^1^H-NMR (DMSO-*d*_6_) δ 7.63 (d, *J* = 8.30 Hz, 2H), 8.13 (m, 3H), 8.92 (d, *J* = 5.86 Hz, 1H), 9.13 (s, 1H) ppm; ^13^C-NMR (DMSO-*d*_6_) δ 111.62, 120.78, 129.26, 130.03, 131.64, 136.69, 142.29, 144.45, 146.46, 146.68, 154.32 ppm; anal. C 46.08, H 2.05, N 19.02% calcd. for C_14_H_8_ClN_5_O_3_S, C 46.48, H 2.23, N 19.36%.

*2-(3-Chloro-4-fluorophenyl)-3-hydroxypyrido[4,3-e][1,2,4]triazino[3,2-c][1,2,4]thiadiazine 6,6-dioxide* (**10**). The mixture of **3** and ethyl 2-(3-chloro-4-fluorophenyl)-2-oxoacetate (0.230 g) gave compound **10** as a white solid powder (0.104 g, 27%), m.p. 389–392 °C; IR (KBr) ν_max_ 3422 (OH), 1642 C=C), 1591 (C=N), 1318, 1182 (SO_2_) cm^−1^; ^1^H-NMR (DMSO-*d*_6_) δ 7.62 (t, *J* = 8.79 Hz, 1H), 8.11–8.22 (m, 2H), 8.23–8.33 (m, 1H), 8.92 (d, *J* = 5.86 Hz, 1H), 9.14 (s, 1H) ppm; ^13^C-NMR (DMSO-*d*_6_) δ 111.76, 117.84, 118.02, 120.45, 120.60, 120.77, 128.55, 130.99, 131.06, 131.91, 142.14, 144.30, 145.56, 146.57, 154.19, 154.45, 158.60, 160.61 ppm; anal. C 44.06, H 1.86, N 18.21% calcd. for C_14_H_7_ClFN_5_O_3_S, C 44.28, H 1.86, N 18.44%.

*3-Hydroxy-2-(4-trifluormethylphenyl)pyrido[4,3-e][1,2,4]triazino[3,2-c][1,2,4]thiadiazine 6,6-dioxide* (**11**). The mixture of **3** and ethyl 2-oxo-2-(4-trifluoromethylphenyl)acetate (0.246 g) gave compound **11** as a white solid powder (0.084 g, 21%), m.p. 378–380 °C; IR (KBr) ν_max_ 3431 (OH), 1642 (C=C), 1589 (C=N), 1382, 1182 (SO_2_) cm^−1^; ^1^H-NMR (DMSO-*d*_6_) δ 7.92 (m, 2H), 8.13 (br. s, 1H), 8.29 (d, *J* = 6.84 Hz, 2H), 8.92 (m, 1H), 9.14 (br. s, 1H) ppm; ^13^C-NMR (DMSO-*d*_6_) δ 111.54, 120.80, 126.04, 130.71, 131.51, 131.77, 135.07, 142.18, 144.35, 146.60, 146.66, 154.17, 154.43 ppm; anal. C 45.25,H 1.81, N 17.42% calcd. for C_15_H_8_F_3_N_5_O_3_S, C 45.57, H 2.04, N 17.72%.

*3-Hydroxy-2-(p-tolyl)pyrido[4,3-e][1,2,4]triazino[3,2-c][1,2,4]thiadiazine 6,6-dioxide* (**12**). The mixture of **3** and ethyl 2-oxo-2-*p*-tolylacetate (0.192 g) gave compound **12** as a white solid powder (0.123 g, 36%), m.p. 359–360 °C; IR (KBr) ν_max_ 3430 (OH), 1633 (C=C), 1587 (C=N), 1321, 1176 (SO_2_) cm^−1^; ^1^H-NMR (DMSO-*d*_6_) δ 2.39 (s, 3H), 7.36 (m, *J* = 7.81 Hz, 2H), 8.03 (m, *J* = 7.81 Hz, 2H), 8.11 (d, *J* = 5.86 Hz, 1H), 8.91 (d, *J* = 5.86 Hz, 1H), 9.12 (s, 1H) ppm; ^13^C-NMR (DMSO-*d*_6_) δ 21.80, 111.56, 120.77, 128.48, 129.74, 129.76, 142.12, 142.30, 144.37, 146.58, 147.27, 154.33 ppm; anal. C 52.9, H 3.07, N 20.36% calcd. for C_15_H_11_N_5_O_3_S, C 52.78, H 3.25, N 20.52%.

*2-(4-Ethylphenyl)-3-hydroxypyrido[4,3-e][1,2,4]triazino[3,2-c][1,2,4]thiadiazine 6,6-dioxide* (**13**). The mixture of **3** and ethyl 2-(4-ethylphenyl)-2-oxoacetate (0.206 g) gave compound **13** as a white solid powder (0.084, 23%), m.p. 375–379 °C; IR (KBr) ν_max_ 3441 (OH), 1633 (C=C), 1587 (C=N), 1321, 1176 (SO_2_) cm^−1^; ^1^H-NMR (DMSO-*d*_6_) δ 1.21 (t, *J* = 7.57 Hz, 3H), 2.69 (q, *J* = 7.32 Hz, 2H), 7.39 (d, *J* = 8.30 Hz, 2H), 8.03 (d, *J* = 8.30 Hz, 2H), 8.11 (d, *J* = 5.86 Hz, 1H), 8.91 (d, *J* = 5.86 Hz, 1H), 9.12 (s, 1H) ppm; ^13^C-NMR (DMSO-*d*_6_) δ 15.76, 28.77, 111.56, 120.95, 128.50, 128.68, 129.87, 142.41, 144.44, 146.34, 147.66, 148.36, 154.15, 154.23 ppm; anal. C 53.81, H 3.53, N 19.41% calcd. for C_16_H_13_N_5_O_3_S, C 54.08, H 3.69, N 19.71%.

*3-Hydroxy-2-(4-propylphenyl)pyrido[4,3-e][1,2,4]triazino[3,2-c][1,2,4]thiadiazine 6,6-dioxide* (**14**). The mixture of **3** and ethyl 2-oxo-2-(4-propylphenyl)acetate (0.220 g) gave compound **14** as a white solid powder (0.092, 25%), m.p. 343–345 °C; IR (KBr) ν_max_ 3417 (OH), 1627 (C=C), 1583 (C=N), 1318, 1167 (SO_2_) cm^−1^; ^1^H-NMR (DMSO-*d*_6_) δ 0.91 (t, *J* = 7.32 Hz, 3H), 1.56–1.58 (m, 2H), 2.63 (t, *J* = 7.57 Hz, 2H), 7.37 (d, *J* = 8.30 Hz, 2H), 8.03 (d, *J* = 8.30 Hz, 2H), 8.11 (d, *J* = 5.86 Hz, 1H), 8.91 (d, *J* = 5.86 Hz, 1H), 9.12 (s, 1H) ppm; ^13^C-NMR (DMSO-*d*_6_) δ 14.30, 24.61, 37.79, 111.57, 120.77, 128.76, 129.15, 129.81, 142.31, 144.37, 146.58, 146.65, 147.38, 154.33 ppm; anal. C 55.27, H 4.09, N 18.86% calcd. for C_17_H_15_N_5_O_3_S, C 55.02, H 3.96, N 18.54%.

*3-Hydroxy-2-(4-isopropylphenyl)pyrido[4,3-e][1,2,4]triazino[3,2-c][1,2,4]thiadiazine 6,6-dioxide* (**15**). The mixture of **3** and ethyl 2-(4-isopropylphenyl)-2-oxoacetate (0.220 g) gave compound **15** as a white solid powder (0.092 g, 25%), m.p. 361–362 °C; IR (KBr) ν_max_ 3430 (OH), 1638 (C=C), 1588 (C=N), 1322, 1182 (SO_2_) cm^−1^; ^1^H-NMR (DMSO-*d*_6_) δ 1.24 (d, *J* = 6.84 Hz, 6H), 2.89–3.03 (m, 1H), 7.42 (d, *J* = 7.81 Hz, 2H), 8.02 (d, *J* = 8.30 Hz, 2H), 8.10 (d, *J* = 5.86 Hz, 1H), 8.91 (d, *J* = 5.86 Hz, 1H), 9.12 (s, 1H) ppm; ^13^C-NMR (DMSO-*d*_6_) δ 24.19, 34.07, 111.57, 120.91, 127.06, 128.82, 129.95, 142.42, 144.49, 146.37, 147.70, 152.86, 154.18, 154.29 ppm; anal. C 54.93, H 3.95, N 18.75% calcd. for C_17_H_15_N_5_O_3_S, C 55.27, H 4.09, N 18.96%.

*3-Hydroxy 2-(4-isobutylphenyl)pyrido[4,3-e][1,2,4]triazino[3,2-c][1,2,4]thiadiazine 6,6-dioxide* (**16**). The mixture of **3** and ethyl 2-(4-isobutylphenyl)-2-oxoacetate (0.234 g) gave compound **16** as a white solid powder (0.085 g, 22%), m.p. 359–361 °C; IR (KBr) ν_max_ 3428 (OH), 1634 (C=C), 1589 (C=N), 1322, 1177 (SO_2_) cm^−1^; ^1^H-NMR (DMSO-*d*_6_) δ 0.88 (d, *J* = 6.35 Hz, 6H), 1.89 (m, 1H), 2.53 (d, *J* = 6.84 Hz, 2H), 7.34 (d, *J* = 7.81 Hz, 2H), 8.03 (d, *J* = 7.81 Hz, 2H), 8.12 (d, *J* = 5.86 Hz, 1H), 8.91 (d, *J* = 5.37 Hz, 1H), 9.12 (s, 1H) ppm; ^13^C-NMR (DMSO-*d*_6_) δ 22.79, 30.23, 45.06, 111.62, 120.76, 128.74, 129.69, 142.37, 144.48, 145.72, 146.42, 147.51, 154.26 ppm; anal. C 55.98, H 4.36, N 18.05% calcd. for C_18_H_17_N_5_O_3_S, C 56.38, H 4.47, N 18.27%.

*2-(4-tert-Butylpheny)-3-hydroxypyrido[4,3-e][1,2,4]triazino[3,2-c][1,2,4]thiadiazine 6,6-dioxide* (**17**). The mixture of **3** and ethyl 2-(4-*tert*-butylphenyl)-2-oxoacetate (0.234 g) gave compound **17** as a white solid powder (0.234 g, 37%), m.p. 385–387 °C; IR (KBr) ν_max_ 3444 (OH), 1638 (C=C), 1589 (C=N), 1324, 1183 (SO_2_) cm^−1^; ^1^H-NMR (DMSO-*d*_6_) δ 1.32 (br. s., 9H), 7.56 (d, *J* = 7.32 Hz, 2H), 8.01 (d, *J* = 7.32 Hz, 2H), 8.10 (d, *J* = 5.37 Hz, 1H), 8.91 (d, *J* = 5.37 Hz, 1H), 9.12 (br. s., 1H) ppm; ^13^C-NMR (DMSO-*d*_6_) δ 31.58, 35.40, 111.53, 120.76, 125.97, 128.51, 129.69, 142.31, 144.41, 146.58, 147.47, 154.30, 154.90 ppm; anal. C 56.10, H 4.46, N 18.14% calcd. for C_18_H_17_N_5_O_3_S, C 56.38, H 4.47, N 18.27%.

*2-(4-Ethoxyphenyl)-3-hydroxypyrido[4,3-e][1,2,4]triazino[3,2-c][1,2,4]thiadiazine 6,6-dioxide* (**18**). The mixture of **3** and ethyl 2-(4-ethoxyphenyl)-2-oxoacetate (0.222 g) gave compound **18** as a white solid powder (0.081 g, 21%), m.p. 321–322 °C; IR (KBr) ν_max_ 3543 (OH), 1626 (C=C), 1581 (C=N), 1316, 1167 (SO_2_) cm^−1^; ^1^H-NMR (DMSO-*d*_6_) δ 1.36 (t, *J* = 7.08 Hz, 3H), 4.12 (q, *J* = 7.16 Hz, 2H), 7.08 (d, *J* = 8.79 Hz, 2H), 8.12 (m, 1H), 8.91 (d, *J* = 5.86 Hz, 1H), 9.11 (s, 1H) ppm; ^13^C-NMR (DMSO-*d*_6_) δ 15.11, 64.25, 111.59, 115.18, 120.95, 123.45, 131.61, 142.44, 144.46, 146.32, 147.03, 154.12, 154.41, 161.87 ppm; anal. C 51.55, H 3.40, N 18.49% calcd. for C_16_H_13_N_5_O_4_S, C 51.75, H 3.53, N 18.86%.

*2-(4-Methoxy-3-methylphenyl)-3-hydroxypyrido[4,3-e][1,2,4]triazino[3,2-c][1,2,4]thiadiazine 6,6-dioxide* (**19**). The mixture of **3** and ethyl 2-(4-methoxy-3-methylphenyl)-2-oxoacetate (0.236 g) gave compound **19** as a white solid powder (0.091 g, 24%), m.p. 318–320 °C; IR (KBr) ν_max_ 3430 (OH), 1632 (C=C), 1591 (C=N), 1327, 1170 (SO_2_) cm^−1^; ^1^H-NMR (DMSO-*d*_6_) δ 2.24 (s, 3H), 3.87 (s, 3H), 7.09 (d, *J* = 8.79 Hz, 1H), 7.93 (s, 1H), 8.06 (dd, *J* = 8.79 Hz, 1H), 8.14 (d, *J* = 5.86 Hz, 1H), 8.92 (d, *J* = 5.86 Hz, 1H), 9.11 (s, 1H) ppm; ^13^C-NMR (DMSO-*d*_6_) δ 16.83, 56.28, 110.69, 111.64, 120.74, 123.08, 126.53, 129.77, 131.42, 142.33, 144.34, 146.53, 146.85, 154.31, 154.43, 160.67 ppm; anal. C 51.88, H 3.45, N 18.50% calcd. for C_16_H_13_N_5_O_4_S, C 51.75, H 3.53, N 18.86%.

*2-(Benzo[d][1,3]dioxol-5-yl)-3-hydroxypyrido[4,3-e][1,2,4]triazino[3,2-c][1,2,4]thiadiazine 6,6-dioxide* (**20**). The mixture of **3** and ethyl 2-(benzo[*d*][1,3]dioxol-5-yl)-2-oxoacetate (0.222 g) gave compound **20** as a white solid powder (0.084 g, 23%), m.p. 319–320 °C; IR (KBr) ν_max_ 3424 (OH), 1632 (C=C), 1588 (C=N), 1321, 1174 (SO_2_) cm^−1^; ^1^H-NMR (DMSO-*d*_6_) δ 6.14 (s, 2H), 7.09 (d, *J* = 8.30 Hz, 1H), 7.64 (s, 1H), 7.76–7.84 (m, 1H), 8.17 (d, *J* = 5.86 Hz, 1H), 8.90 (d, *J* = 6.35 Hz, 1H), 9.11 (s, 1H) ppm; ^13^C-NMR (DMSO-*d*_6_) δ 102.50, 108.92, 109.27, 111.74, 120.66, 124.88, 125.43, 142.26, 144.29, 146.39, 146.59, 148.11, 150.58, 154.30 ppm; anal. C 48.41, H 2.36, N 18.51% calcd. for C_15_H_9_N_5_O_3_S, C 48.52, H 2.44, N 18.86%.

#### 4.2.3. General Procedure for the Preparation of 3-Phenylpyrido[4,3-*e*][1,2,4]triazino[3,2-*c*][1,2,4]-thiadiazine 6,6-dioxides **21**–**28**

The mixture of **3** (2.5 mmol) and the appropriate phenylglyoxal hydrate (2.5 mmol) in glacial acetic acid (6 mL) was stirred at reflux for 23–31 h. After standing at room temperature, the precipi-tate was filtered off and dried. A final purification was described below.

*3-Phenylpyrido[4,3-e][1,2,4]triazino[3,2-c][1,2,4]thiadiazine 6,6-dioxide* (**21**). The mixture of **3** and 1-phenyl-2,2-dihydroxyethanone (0.380 g) gave compound **21** as a dark yellow solid powder. The product was purified by extraction of contaminants with boiling ethanol (0.437 g, 56%), m.p. 259–261 °C; IR (KBr) ν_max_ 1575 (C=N), 1315, 1123 (SO_2_) cm^−1^; ^1^H-NMR (DMSO-*d*_6_) δ 7.69 (m, 2H); 7.80 (m, 1H); 8.15 (d, *J* = 4.88 Hz, 1H); 8.43 (d, *J* = 7.32 Hz, 2H); 9.00 (d, *J* = 5.37 Hz, 1H); 9.24 (br. s, 1H); 9.51 (br. s, 1H) ppm; ^13^C-NMR (DMSO-*d*_6_) δ 111.95, 120.76, 129.91, 130.20, 132.24, 135.58, 137.37, 142.24, 146.70, 146.87, 154.19, 162.63 ppm; anal. C 53.78, H 2.94, N 22.25% calcd. for C_14_H_9_N_5_O_3_S, C 54.01, H 2.91, N 22.50%.

*3-(3-Fluorophenyl)pyrido[4,3-e][1,2,4]triazino[3,2-c][1,2,4]thiadiazine 6,6-dioxide* (**22**). The mixture of **3** and 1-(3-fluorophenyl)-2,2-dihydroxyethanone (0.425 g) gave compound **22** as a white solid powder. The product was purified by extraction of contaminants with boiling benzene and crystallization from acetonitrile (0.172 g, 20%), m.p. 264–266 °C; IR (KBr) ν_max_ 1580 (C=N), 1306, 1119 (SO_2_) cm^−1^; ^1^H-NMR (DMSO-*d*_6_) δ 7.68 (m, 1H), 7.75 (m, 1H), 8.18 (d, 1H), 8.28 (m, 2H), 9.02 (d, 1H), 9.24 (s, 1H), 9.51 (s, 1H) ppm; ^13^C-NMR (DMSO-*d*_6_) δ 112.17, 116.38, 116.57, 120.89, 122.46, 122.63, 126.26, 126.28, 132.57, 132.64, 134.68, 134.74, 137.50, 142.36, 146.71, 147.09, 154.45, 161.91, 162.25, 164.21 ppm; anal. C 50.83, H 2.49, N 20.92% calcd. for C_14_H_8_FN_5_O_2_S, C 51.06, H 2.45, N 21.27%.

*3-[4-(Trifluoromethyl)phenyl]pyrido[4,3-e][1,2,4]triazino[3,2-c][1,2,4]thiadiazine 6,6-dioxide* (**23**). The mixture of **3** and 2,2-dihydroxy-1-[4-(trifluoromethyl)phenyl]ethanone (0.550 g) gave compound **23** as a white solid powder. The product was purified by extraction of contaminants with boiling benzene and crystallization from acetonitrile (0.151 g, 15%), m.p. 292–295 °C; IR (KBr) ν_max_ 1594 (C=N), 1324, 1117 (SO_2_) cm^−1^; ^1^H-NMR (DMSO-*d*_6_) δ 8.07–8.18 (m, 3H), 8.60–8.62 (m, 2H), 9.02–9.03 (m, 1H), 9.25 (s, 1H), 9.56 (s, 1H) ppm; ^13^C-NMR (DMSO-*d*_6_) δ 112.20, 120.89, 127.13, 127.15, 130.80, 136.16, 137.63, 142.33, 146.73, 147.12, 154.50, 162.03 ppm; anal. C 47.65, H 2.13,N 18.24% calcd. for C_15_H_8_F_3_N_5_O_2_S, C 47.50, H 2.13, N 18.46%.

*3-(3,4-Difluorophenyl)pyrido[4,3-e][1,2,4]triazino[3,2-c][1,2,4]thiadiazine 6,6-dioxide* (**24**). The mixture of **3** and 1-(3,4-difluorophenyl)-2,2-dihydroxyethanone (0.470 g) gave compound **24** as a white solid powder. The product was purified by extraction of contaminants with boiling ethanol (0.197 g, 24%), m.p. 309–311 °C; IR (KBr) ν_max_ 1582 (C=N), 1313, 1120 (SO_2_) cm^−1^; ^1^H-NMR (DMSO-*d*_6_)δ 7.70 (t, *J* = 7.57 Hz, 1H), 8.18 (d, *J* = 5.86 Hz, 1H), 8.30 (d, *J* = 8.30 Hz, 1H), 8.44 (d, *J* = 10.74 Hz, 1H), 9.02 (d, *J* = 5.86 Hz, 1H), 9.25 (s, 1H), 9.50 (s, 1H) ppm; ^13^C-NMR (DMSO-*d*_6_) δ 110.98, 113.24, 115.50, 122.52, 126.23, 131.58, 131.72, 131.98, 132.04, 133.14, 136.99, 142.11, 144.45, 146.69, 147.73, 160.26 ppm; anal. C 48.12, H 2.13, N 19.88% calcd. for C_14_H_7_F_2_N_5_O_2_S, C 48.42, H 2.03, N 20.17%.

*3-(3-Methoxyphenyl)pyrido[4,3-e][1,2,4]triazino[3,2-c][1,2,4]thiadiazine 6,6-dioxide* (**25**). The mixture of **3** and 2,2-dihydroxy-1-(3-methoxyphenyl)ethanone (0.455 g) gave compound **25** as a yellow solid powder. The product was purified by extraction of contaminants with boiling ethanol (0.400 g, 47%), m.p. 270–272 °C; IR (KBr) ν_max_ 1586 (C=N), 1299, 1164 (SO_2_) cm^−1^; ^1^H-NMR (DMSO-*d*_6_) δ 3.90 (s, 3H), 7.39 (m, 1H), 7.62 (t, 1H), 7.91 (s, 1H), 8.03 (m, 1H), 8,16 (m, 1H), 9.02 (m, 1H), 9.23 (s, 1H), 9.52 (s, 1H) ppm; ^13^C-NMR (DMSO-*d*_6_) δ 55.65, 111.50, 113.53, 120.25, 121.34, 122.00, 131.00, 133.08, 137.00, 141.74, 146.14, 146.80, 153.75, 160.01, 161.90 ppm; anal. C 52.44, H 3.07, N 20.29% calcd. for C_15_H_11_N_5_O_3_S, C 52.78, H 3.25, N 20.52%.

*3-(4-Methoxyphenyl)pyrido[4,3-e][1,2,4]triazino[3,2-c][1,2,4]thiadiazine 6,6-dioxide* (**26**). The mixture of **3** and 2,2-dihydroxy-1-(4-methoxyphenyl)ethanone (0.455 g) gave compound **26** as a yellow solid powder. The product was purified by crystallization from acetonitrile (0.169 g, 25%), m.p. 276–278 °C; IR (KBr) ν_max_ 1579 (C=N), 1301, 1164 (SO_2_) cm^−1^; ^1^H-NMR (DMSO-*d*_6_) δ 3.93 (s, 3H), 7.25 (d, 2H), 8.12–8.13 (m, 2H), 8.44–8.46 (m, 2H), 8.98–8.99 (m, 1H), 9.20 (s, 1H), 9.46 (s, 1H) ppm; ^13^C-NMR (DMSO-*d*_6_) δ 56.72, 112.00, 116.10, 121.03, 124.72, 132.75, 137.45, 142.50, 146.94, 154.25, 161.56, 165.93 ppm; anal. C 52.65, H 3.03, N 20.34% calcd. for C_15_H_11_N_5_O_3_S, C 52.78, H 3.25, N 20.52%.

*3-(3,4-Dimethoxyphenyl)pyrido[4,3-e][1,2,4]triazino[3,2-c][1,2,4]thiadiazine 6,6-dioxide* (**27**). The mixture of **3** and 1-(3,4-dimethoxyphenyl)-2,2-dihydroxyethanone (0.530 g) gave compound **27** as an orange solid powder. The product was purified by extraction of contaminants with boiling ethanol (0.490 g, 53%), m.p. 264–265 °C; IR (KBr) ν_max_ 1566 (C=N), 1302, 1111(SO_2_) cm^−1^; ^1^H-NMR (DMSO-*d*_6_) δ 3.91 (s, 3H), 3.94 (s, 3H), 7.27–7.28 (d, 1H), 7.86–7.90 (m, 1H), 8.12–8.13 (s, 1H), 8.17–8.21 (m, 1H), 8.98–8.99 (m,1H), 9.20 (s, 1H), 9.51 (s, 1H) ppm; ^13^C-NMR (DMSO-*d*_6_) δ 55.37, 55.69, 111.24, 111.54, 116.03, 122.62, 123.62, 127.69, 128.21, 137.73, 141.83, 144.37, 147.40, 147.95, 149.98, 158.12 ppm; anal. C 51.37H 3.18, N 18.52% calcd. for C_16_H_13_N_5_O_4_S, C 51.75, H 3.53, N 18.86%.

*3-(3,4,5-Trimethoxyphenyl)pyrido[4,3-e][1,2,4]triazino[3,2-c][1,2,4]thiadiazine 6,6-dioxide* (**28**). The mixture of **3** and 2,2-dihydroxy-1-(3,4,5-trimethoxyphenyl)ethanone (0.605 g) gave compound **28** as a red solid powder. The product was purified by extraction of contaminants with boiling acetonitrile (0.283 g, 28%), m.p. 257–259 °C; IR (KBr) ν_max_ 1572 (C=N), 1316, 1114 (SO_2_) cm^−1^; ^1^H-NMR (DMSO-*d*_6_) δ 3.84 (s, 3H), 3.94 (s, 6H), 7.73 (s, 2H), 8.15–8.16 (m, 1H), 9.00–9.01 (m, 1H), 9.22 (s, 1H), 9.59 (s, 1H) ppm; ^13^C-NMR (DMSO-*d*_6_) δ 55.74, 61.12, 108.28, 116.12, 122.61, 125.65, 137.64,141.92, 144.52, 146.15, 147.12, 147.81, 153.22 ppm; anal. C 50.63, H 3.65, N 17.30% calcd. for C_17_H_15_N_5_O_5_S, C 50.87, H 3.77, N 17.45%.

### 4.3. Biological Methods

#### Cell Culture and Cell Viability Assay

All chemicals, if not stated otherwise, were obtained from Sigma-Aldrich (St. Louis, MO, USA). The MCF-7 cell line was purchased from Cell Lines Services (Eppelheim, Germany), the HeLa and HCT-116 cell lines were obtained from the Department of Microbiology, Tumor and Cell Biology, Karolinska Institute (Stockholm, Sweden). Cells were cultured in in Dulbecco’s modified Eagle’s medium (DMEM) supplemented with 10% fetal bovine serum, 2 mM glutamine, 100 units/mL penicillin, and 100 μg/mL streptomycin. Cultures were maintained in a humidified atmosphere with 5% CO_2_ at 37 °C in an incubator (Hera Cell, Heraeus, Langenselbold, Germany). Cell viability was determined using the MTT (3-(4,5-dimethylthiazol-2-yl)-2,5-diphenyl-tetrazoliumbromide) assay. Cells were seeded in 96-well plates at a density of 5 × 10^3^ cells/well and treated for 72 h with the examined compounds in the concentration range 1–100 μM. Cisplatin was used as a control compound and was examined in the concentration range 0.01–10 μM. Following treatment, MTT (0.5 mg/mL) was added to the medium and cells were further incubated for 2 h at 37 °C. Cells were lysed with DMSO and the absorbance of the formazan solution was measured at 550 nm with a plate reader (1420 multilabel counter, Victor, Jügesheim, Germany). The optical density of the formazan solution was measured at 550 nm with a plate reader (Victor 1420 multilabel counter). The experiment was performed in triplicate. Results are expressed as IC_50_ values. Values are expressed as the mean ± SD of at least three independent experiments.

## 5. Conclusions

We have developed methods for the synthesis of novel heterocyclic pyrido[4,3-*e*][1,2,4]triazino[3,2-*c*][1,2,4]thiadiazine 6,6-dioxide ring system derivatives **4**–**28** by the reactions of 3-amino-2-(4-thioxo-1,4-dihydropyridine-3-sulfonyl)guanidine **3** with either 2-oxo-alkanoic acids and their esters, or phenylglyoxylate hydrate in glacial acetic acid. We found that some of 3-phenylpyrido[4,3-*e*][1,2,4]triazino[3,2-*c*][1,2,4]thiadiazine 6,6-dioxides **22**, **23**, **25** and **28** exhibited structure-dependent reasonable or moderate anticancer activity with IC_50_ = 25–57 μM, against MCF-7 (breast) and HeLa (cervical cancer), and distinct activity and selectivity towards HCT-116 colon cancer cell line, particularly for compounds **22**, **23** and **25**–**28** with IC_50_ values in the range of 9–47 μM. These promising results encouraged us for further synthesis and biological evaluations of related 3-aryl substituted derivatives, along with potency optimisation, and will be published elsewhere.

## Supplementary Materials

Supplementary materials can be accessed at: http://www.mdpi.com/1420-3049/21/1/41/s1.
