# Supplementary material for: Synthesis of Novel Pyrido[4,3-e][1,2,4]triazino[3,2-c][1,2,4]thiadiazine 6,6-dioxide Derivatives with Potential Anticancer Activity
Source: Molecules. 2015 Dec 29;21(1):41. doi: 10.3390/molecules21010041 (PMC6273831; doi:10.3390/molecules21010041)
Supplement: Supplementary file 1 [file molecules-21-00041-s001.pdf]

Supplementary Materials: Synthesis of Novel Pyrido[4,3-*e*][1,2,4]triazino[3,2-*c*][1,2,4]thiadiazine 6,6-Dioxide Derivatives with Potential Anticancer Activity

Jarosław Sławiński [1,*], Aleksandra Grzonek [1], Beata Żołnowska [1] and Anna Kawiak [2,3]

Compound	Page
Compound **5** ^{1}H-NMR (500 MHz, DMSO-d_6)	S2
Compound **5** ^{13}C-NMR (125 MHz, DMSO-d_6)	S2
Compound **5** IR	S3
Compound **6** ^{1}H-NMR (500 MHz, DMSO-d_6)	S3
Compound **6** ^{13}C-NMR (125 MHz, DMSO-d_6)	S4
Compound **6** IR	S4
Compound **8** ^{1}H-NMR (500 MHz, DMSO-d_6)	S5
Compound **8** ^{13}C-NMR (125 MHz, DMSO-d_6)	S5
Compound **8** IR	S6
Compound **17** ^{1}H-NMR (500 MHz, DMSO-d_6)	S6
Compound **17** ^{13}C-NMR (125 MHz, DMSO-d_6)	S7
Compound **17** IR	S7
Compound **19** ^{1}H-NMR (500 MHz, DMSO-d_6)	S8
Compound **19** ^{13}C-NMR (125 MHz, DMSO-d_6)	S8
Compound **19** IR	S9
Compound **22** ^{1}H-NMR (500 MHz, DMSO-d_6)	S9
Compound **22** ^{13}C-NMR (125 MHz, DMSO-d_6)	S10
Compound **22** IR	S10
Compound **26** ^{1}H-NMR (500 MHz, DMSO-d_6)	S11
Compound **26** ^{13}C-NMR (125 MHz, DMSO-d_6)	S11
Compound **26** IR	S12
Compound **7** COSY	S12
Compound **7** ROESY	S13
Compound **7** HMBC	S13
Compound **7** LC-MS	S14
Compound **25** COSY	S14
Compound **25** ROESY	S15
Compound **25** HMBC	S15

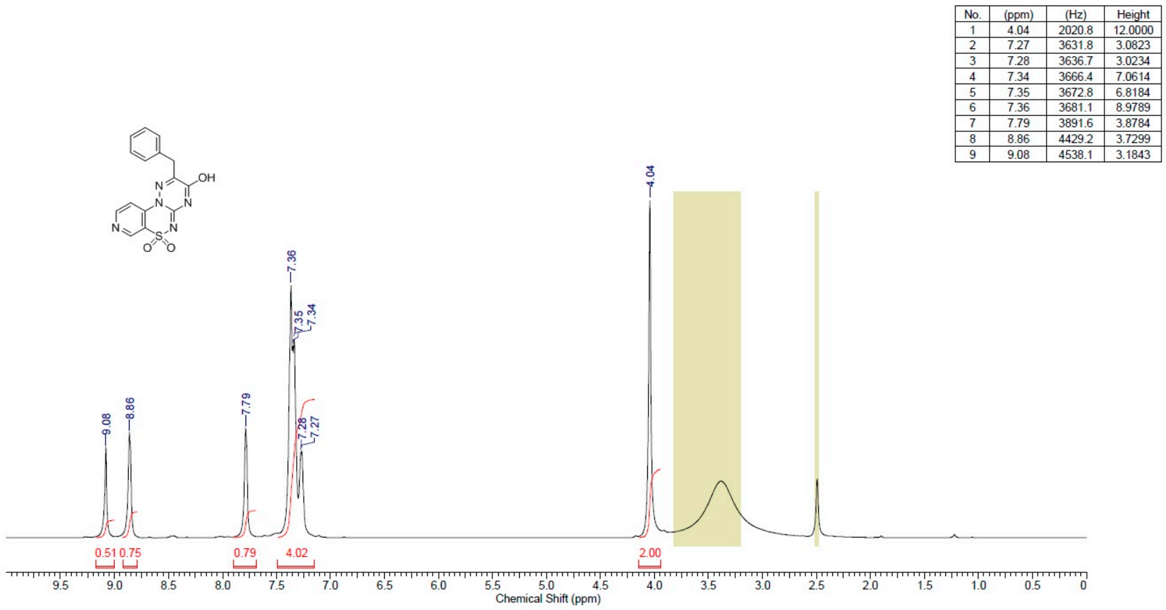

No.	(ppm)	(Hz)	Height
1	4.04	2020.8	12.0000
2	7.27	3631.8	3.0823
3	7.28	3636.7	3.0234
4	7.34	3666.4	7.0614
5	7.35	3672.8	6.8184
6	7.36	3681.1	8.9789
7	7.79	3891.6	3.8784
8	8.86	4429.2	3.7299
9	9.08	4538.1	3.1843

Figure S1. ^{1}H-NMR (500 MHz, DMSO-d_6) spectrum of compound **5**.

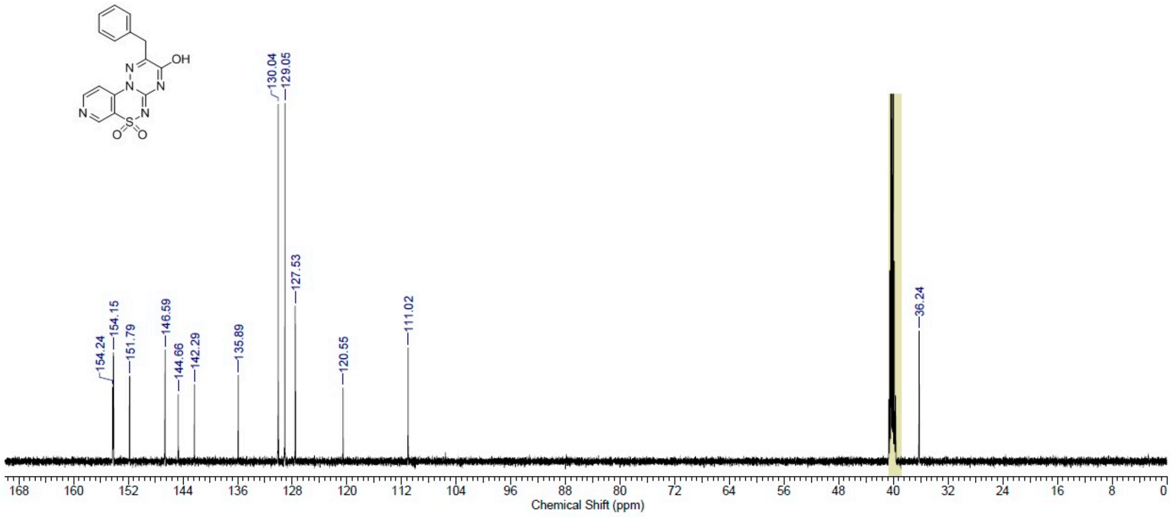

No.	(ppm)	(Hz)	Height	No.	(ppm)	(Hz)	Height	No.	(ppm)	(Hz)	Height	No.	(ppm)	(Hz)	Height	No.	(ppm)	(Hz)	Height
1	36.24	4554.9	4.3693	4	127.53	16029.3	5.2107	7	135.89	17080.1	2.8958	10	146.59	18425.5	3.7447	13	154.24	19386.3	2.4879
2	111.02	13954.1	3.8162	5	129.05	16220.3	12.0000	8	142.29	17885.0	2.5864	11	151.79	19078.9	2.8698				
3	120.55	15152.0	2.4724	6	130.04	16344.6	11.9621	9	144.66	18182.2	2.2468	12	154.15	19374.9	3.6599				

Figure S2. ^{13}C-NMR (125 MHz, DMSO-d_6) spectrum of compound **5**.

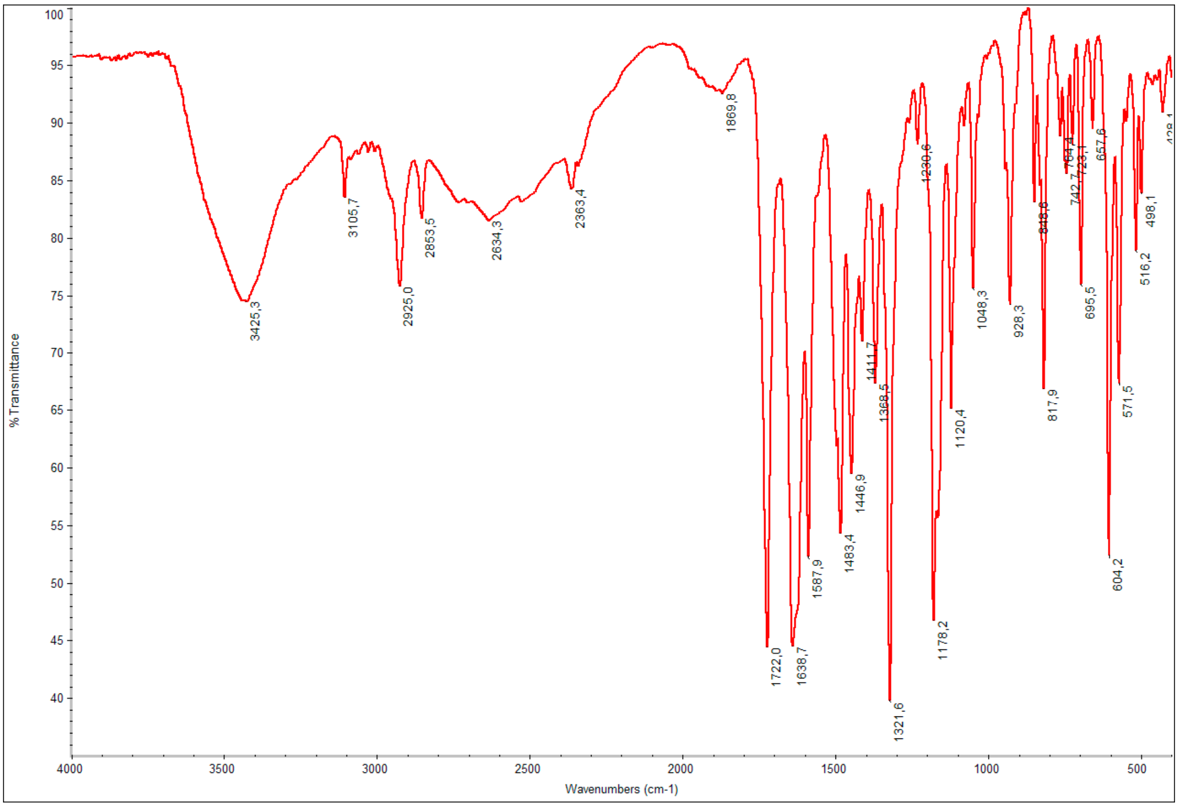

Figure S3. IR spectrum of compound **5**.

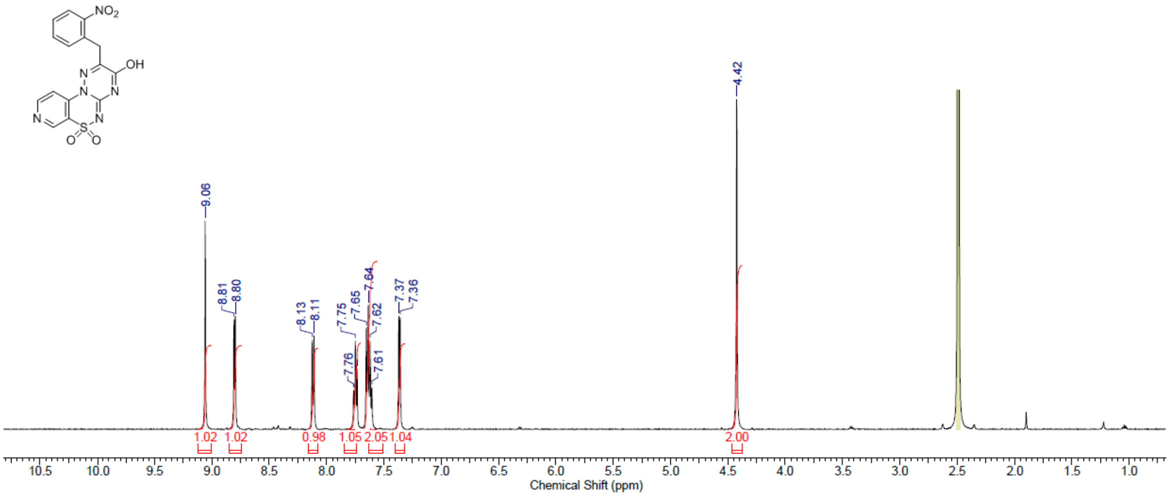

No.	(ppm)	(Hz)	Height	No.	(ppm)	(Hz)	Height	No.	(ppm)	(Hz)	Height	No.	(ppm)	(Hz)	Height	No.	(ppm)	(Hz)	Height
1	4.42	2210.8	0.3891	4	7.61	3801.7	0.0495	7	7.65	3825.1	0.1187	10	7.76	3880.8	0.0459	13	8.80	4396.5	0.1326
2	7.36	3678.2	0.1315	5	7.62	3809.0	0.1038	8	7.74	3866.2	0.0707	11	8.11	4053.7	0.1102	14	8.81	4402.3	0.1290
3	7.37	3684.0	0.1333	6	7.64	3817.3	0.1460	9	7.75	3873.5	0.1038	12	8.13	4061.5	0.1051	15	9.06	4528.3	0.2457

Figure S4. ^{1}H-NMR (500 MHz, DMSO-d_6) spectrum of compound **6**.

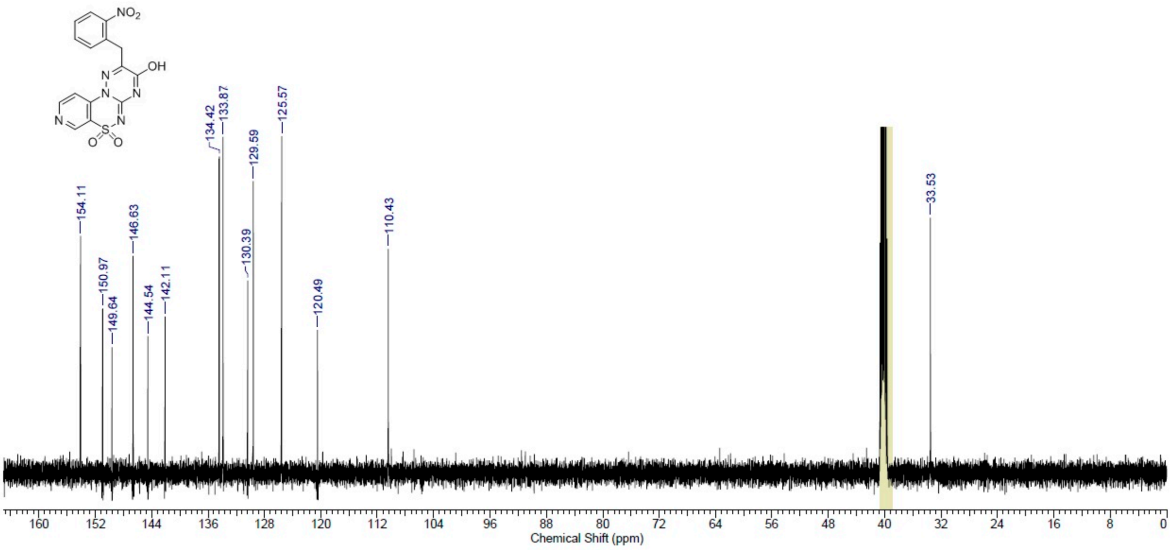

No.	(ppm)	(Hz)	Height
1	33.53	4214.2	9.0995
2	110.43	13880.3	7.9795
3	120.49	15144.5	5.0986
4	125.57	15782.5	12.0000
5	129.59	16288.8	10.3978
6	130.39	16388.9	6.8538
7	133.87	16826.3	11.9764
8	134.42	16895.7	11.2804
9	142.11	17862.1	5.5867
10	144.54	18167.8	4.8874
11	146.63	18430.3	7.7436
12	149.64	18808.4	4.4812
13	150.97	18975.7	5.8638
14	154.11	19370.5	8.4484

Figure S5. ^{13}C-NMR (125 MHz, DMSO-d_6) spectrum of compound **6**.

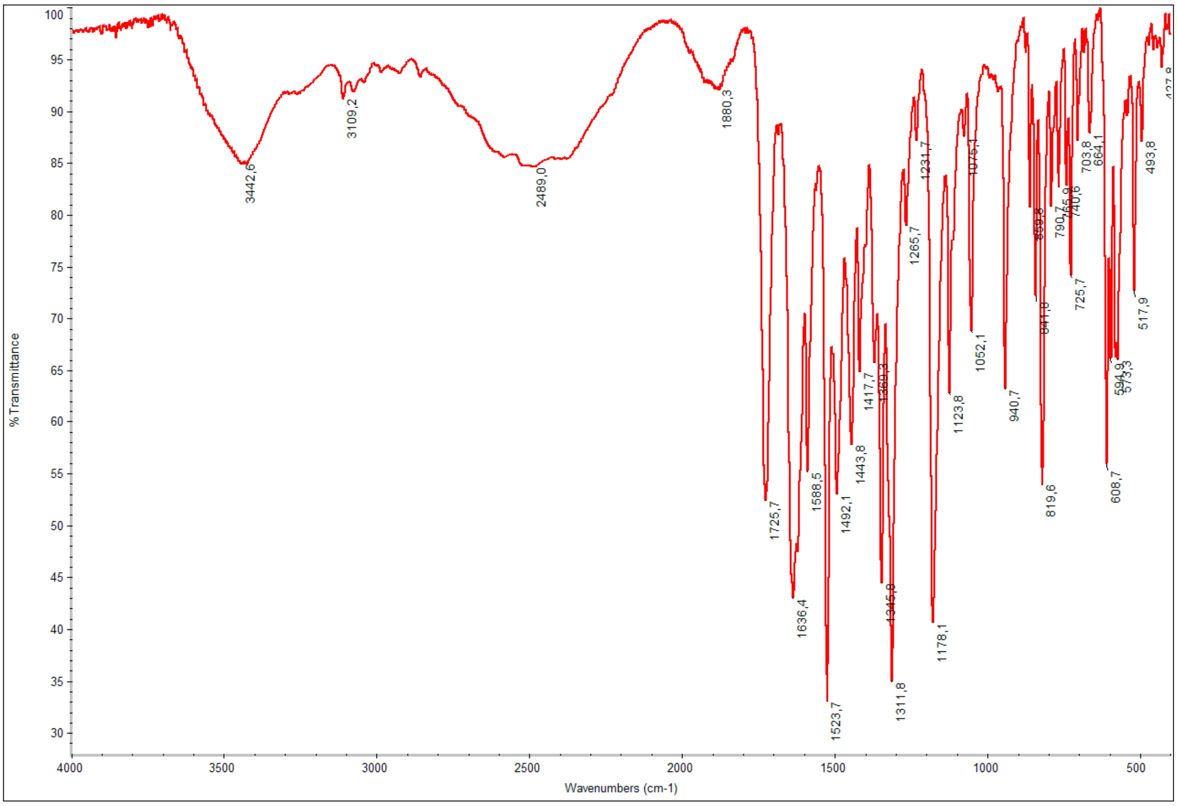

Figure S6. IR spectrum of compound **6**.

No.	(ppm)	(Hz)	Height
1	2.05	1025.7	5.9388
2	2.92	1461.7	11.8419
3	2.94	1468.1	12.0000
4	7.37	3683.0	3.1479
5	7.38	3689.4	3.5419
6	7.88	3936.5	3.2354
7	7.89	3942.8	3.4492
8	7.94	3967.2	5.0188
9	8.10	4048.3	4.1767
10	8.92	4456.0	3.9850
11	9.11	4555.2	4.2403

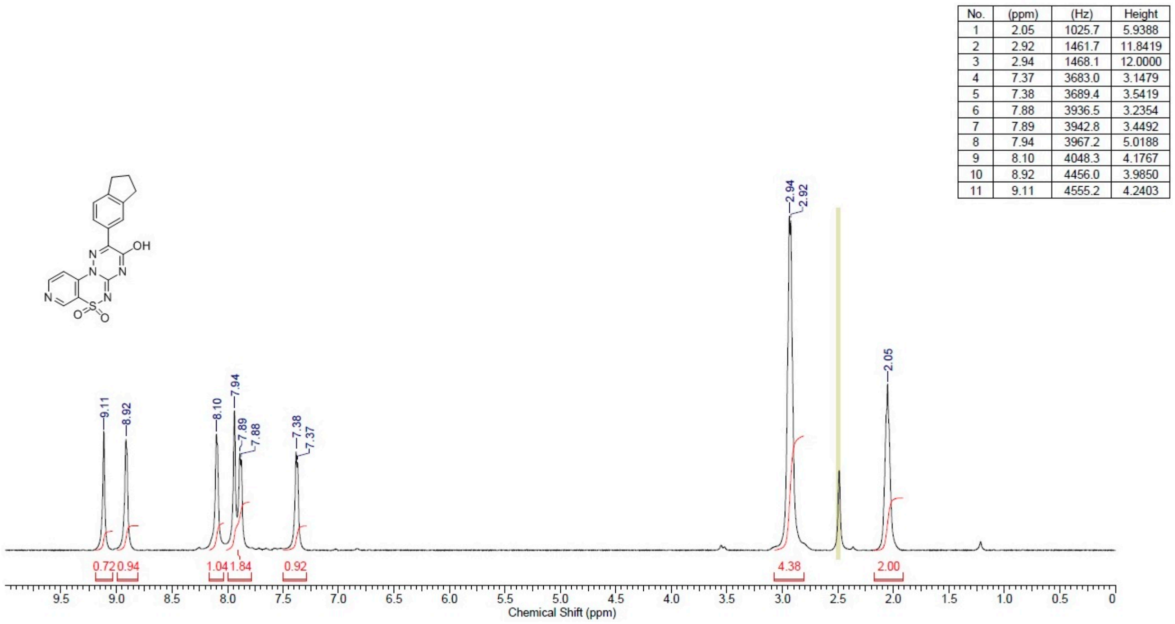

Figure S7. ^{1}H-NMR (500 MHz, DMSO-d_6) spectrum of compound **8**.

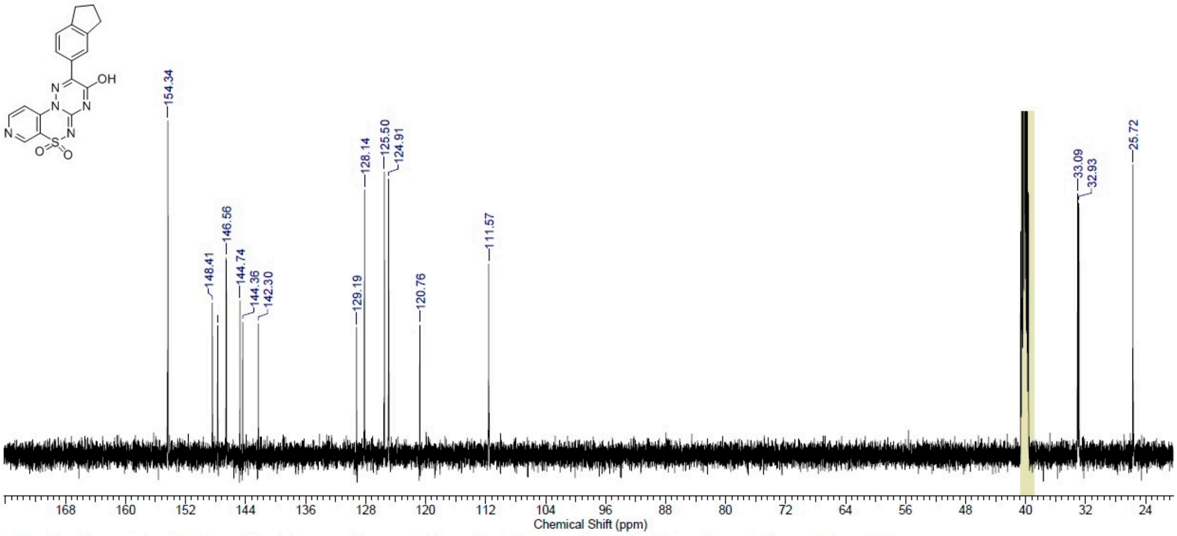

No.	(ppm)	(Hz)	Height	No.	(ppm)	(Hz)	Height	No.	(ppm)	(Hz)	Height	No.	(ppm)	(Hz)	Height
1	25.72	3232.4	10.4236	5	120.76	15178.3	4.6597	9	129.19	16238.3	4.5850	13	146.56	18421.6	7.0891
2	32.93	4139.1	9.0496	6	124.91	15700.4	9.9045	10	142.30	17885.8	4.7204	14	147.70	18564.7	4.6585
3	33.09	4159.8	9.3905	7	125.50	15773.7	10.1661	11	144.36	18144.9	4.7707	15	148.41	18654.3	5.4582
4	111.57	14023.9	6.8565	8	128.14	16106.6	9.5282	12	144.74	18192.8	5.5363	16	154.34	19399.0	12.0000

Figure S8. ^{13}C-NMR (125 MHz, DMSO-d_6) spectrum of compound **8**.

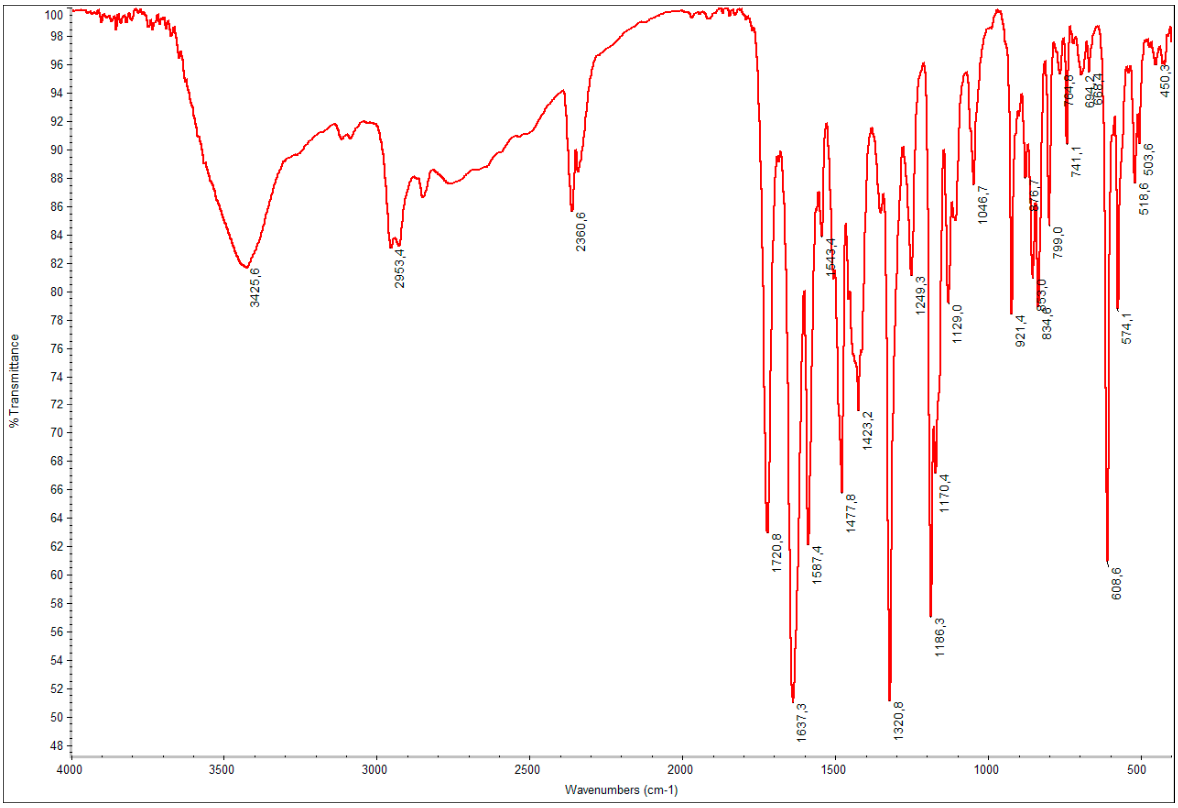

Figure S9. IR spectrum of compound **8.**

No.	(ppm)	(Hz)	Height
1	1.32	660.9	12.0000
2	7.56	3779.2	1.3736
3	7.58	3786.6	1.3280
4	8.01	4005.3	1.3279
5	8.03	4012.7	1.1563
6	8.10	4046.8	0.8155
7	8.11	4052.2	0.7116
8	8.91	4451.2	0.7960
9	8.92	4456.5	0.6663
10	9.12	4560.1	0.8516

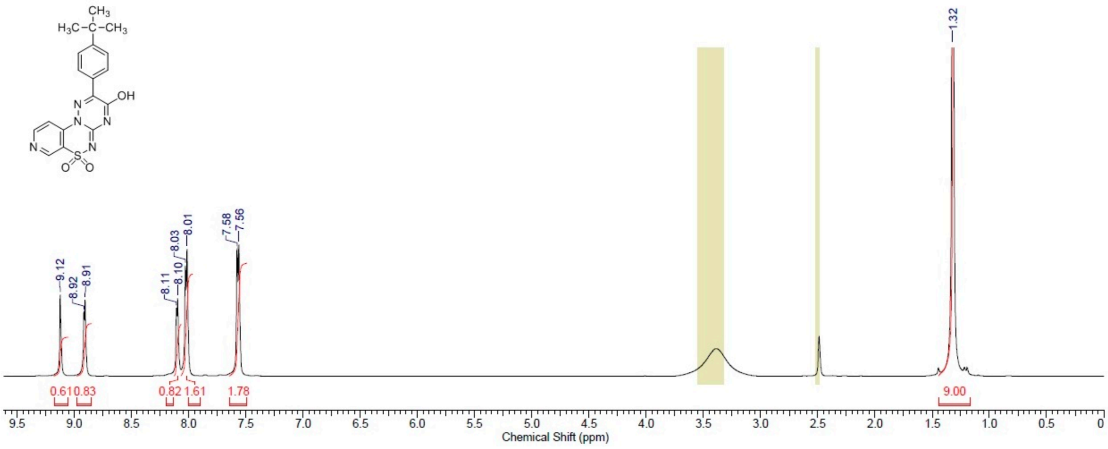

Figure S10. ^{1}H-NMR (500 MHz, DMSO-*d*6) spectrum of compound **17**.

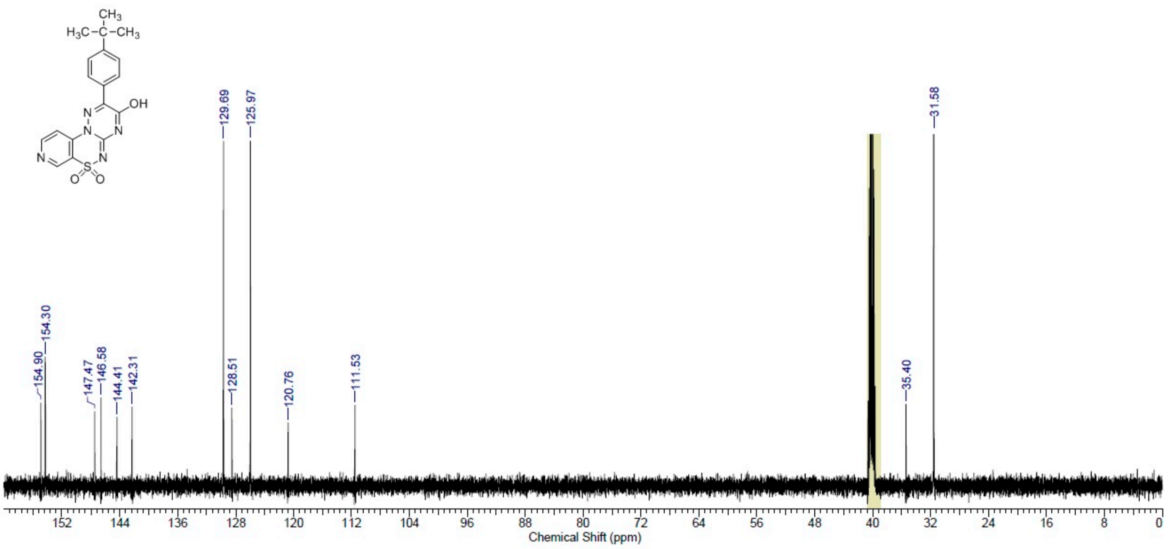

No.	(ppm)	(Hz)	Height
1	31.58	3969.2	12.0000
2	35.40	4449.6	1.6555
3	111.53	14018.2	1.6362
4	120.76	15178.7	1.2834
5	125.97	15833.9	7.0010
6	128.51	16153.1	1.5884
7	129.69	16300.7	6.9912
8	142.31	17887.6	1.6049
9	144.41	18150.6	1.4051
10	146.58	18424.2	1.7872
11	147.47	18535.7	1.5138
12	154.30	19393.7	2.6193
13	154.90	19469.3	1.6869

Figure S11. ^{13}C-NMR (125 MHz, DMSO-d_6) spectrum of compound **17**.

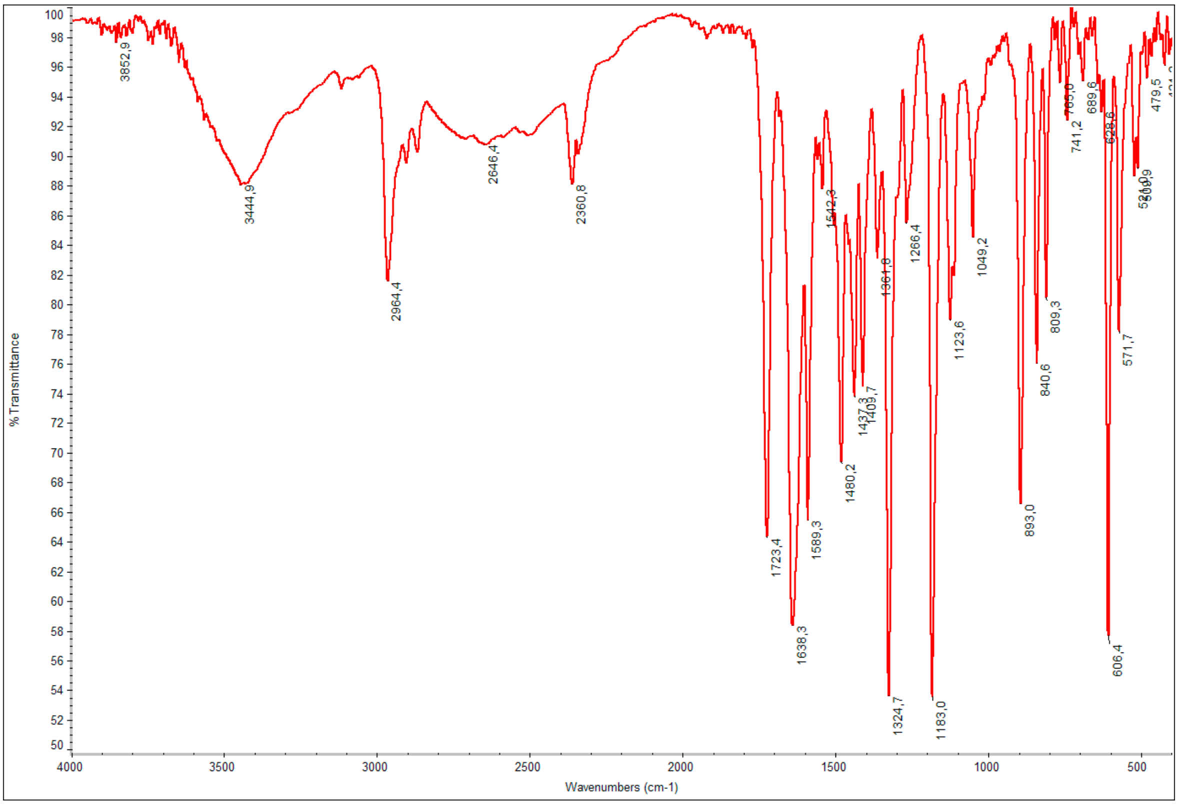

Figure S12. IR spectrum of compound **17**.

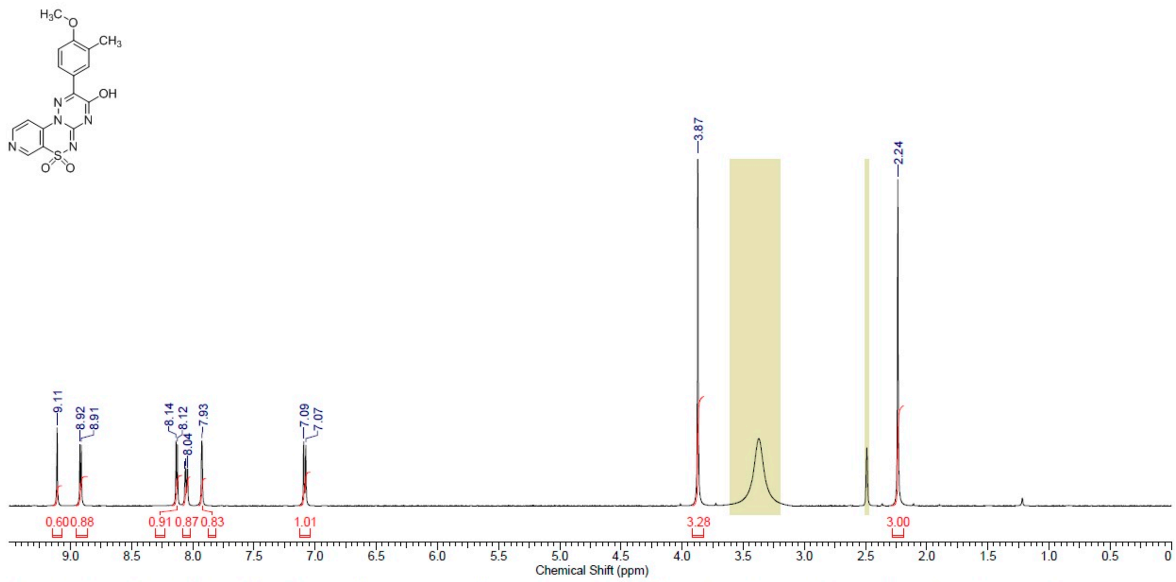

No.	(ppm)	(Hz)	Height	No.	(ppm)	(Hz)	Height	No.	(ppm)	(Hz)	Height	No.	(ppm)	(Hz)	Height	No.	(ppm)	(Hz)	Height
1	2.24	1117.5	9.6610	4	7.09	3544.9	1.9075	7	8.04	4021.0	1.0472	10	8.12	4061.0	1.8159	13	8.92	4459.5	1.8148
2	3.87	1934.4	12.0000	5	7.93	3961.4	1.9189	8	8.06	4027.8	1.0906	11	8.14	4066.9	1.9278	14	9.11	4552.2	2.3145
3	7.07	3536.1	1.8022	6	8.04	4019.0	1.0910	9	8.06	4029.7	1.0126	12	8.91	4453.6	1.8109				

Figure S13. ^{1}H-NMR (500 MHz, DMSO-d_6) spectrum of compound **19**.

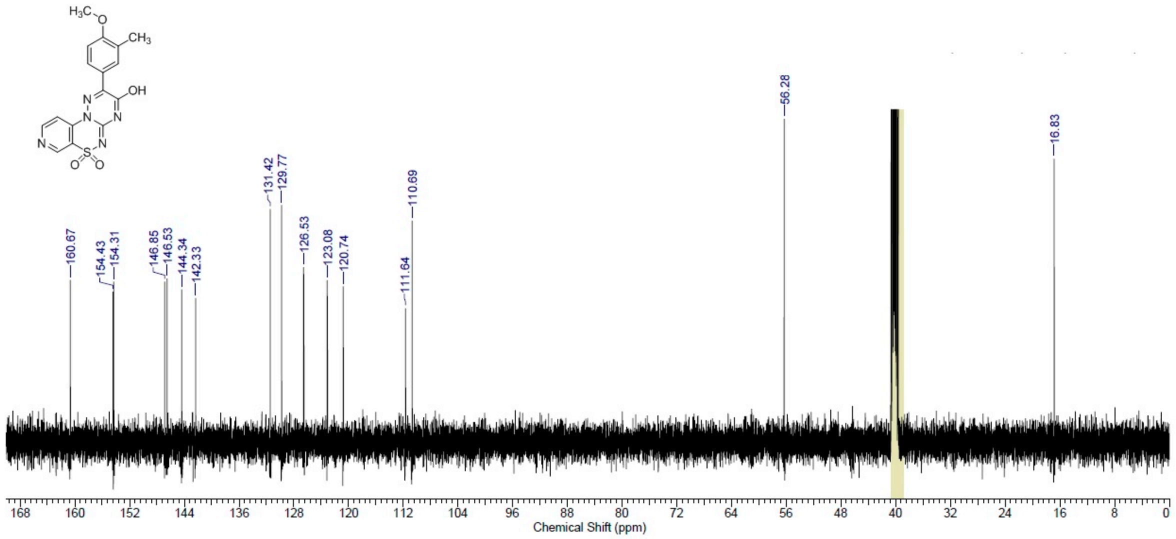

No.	(ppm)	(Hz)	Height	No.	(ppm)	(Hz)	Height	No.	(ppm)	(Hz)	Height	No.	(ppm)	(Hz)	Height
1	16.83	2114.8	10.5148	5	120.74	15176.5	5.7798	9	131.42	16518.0	8.6425	13	146.85	18457.6	5.9628
2	56.28	7074.6	12.0000	6	123.08	15470.3	6.0118	10	142.33	17889.4	5.3473	14	154.31	19395.1	5.9536
3	110.69	13913.2	8.2186	7	126.53	15903.7	6.5014	11	144.34	18142.3	5.6757	15	154.43	19410.0	5.5777
4	111.64	14032.7	4.9774	8	129.77	16311.2	8.7959	12	146.53	18417.2	6.0812	16	160.67	20194.7	6.0186

Figure S14. ^{13}C-NMR (125 MHz, DMSO-d_6) spectrum of compound **19**.

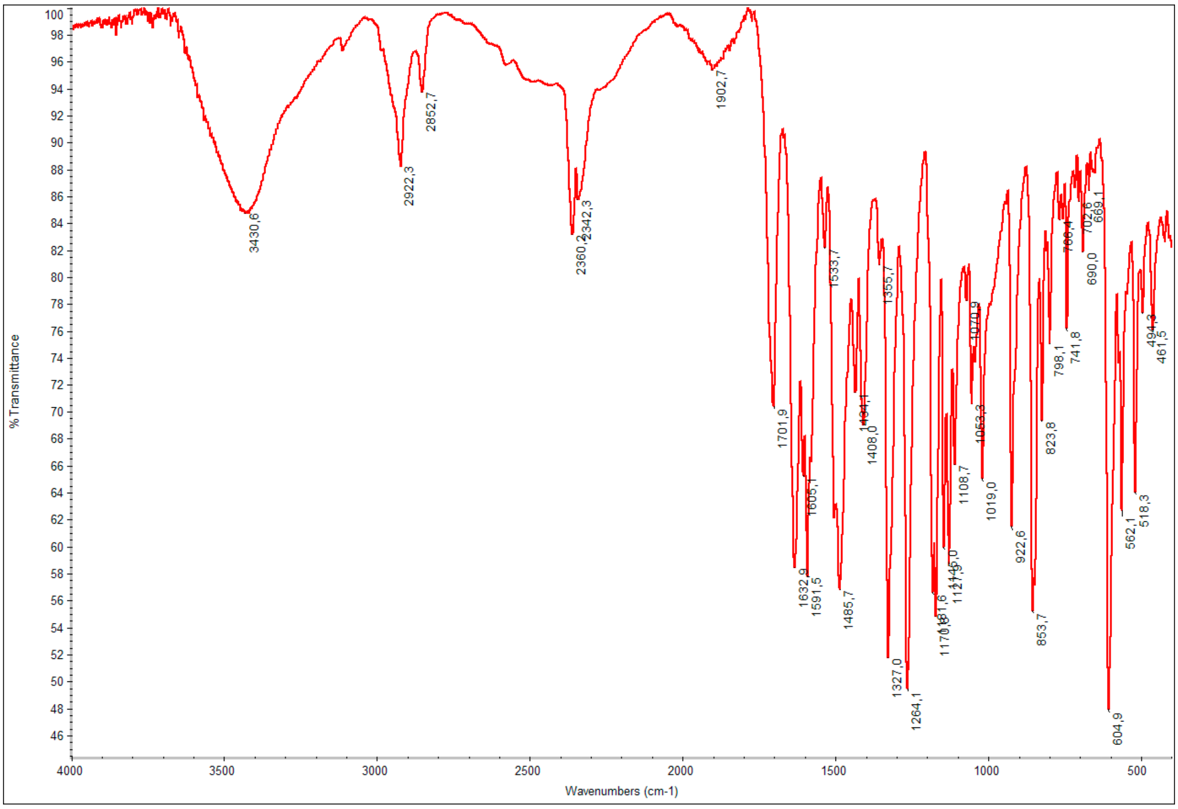

Figure S15. IR spectrum of compound **19**.

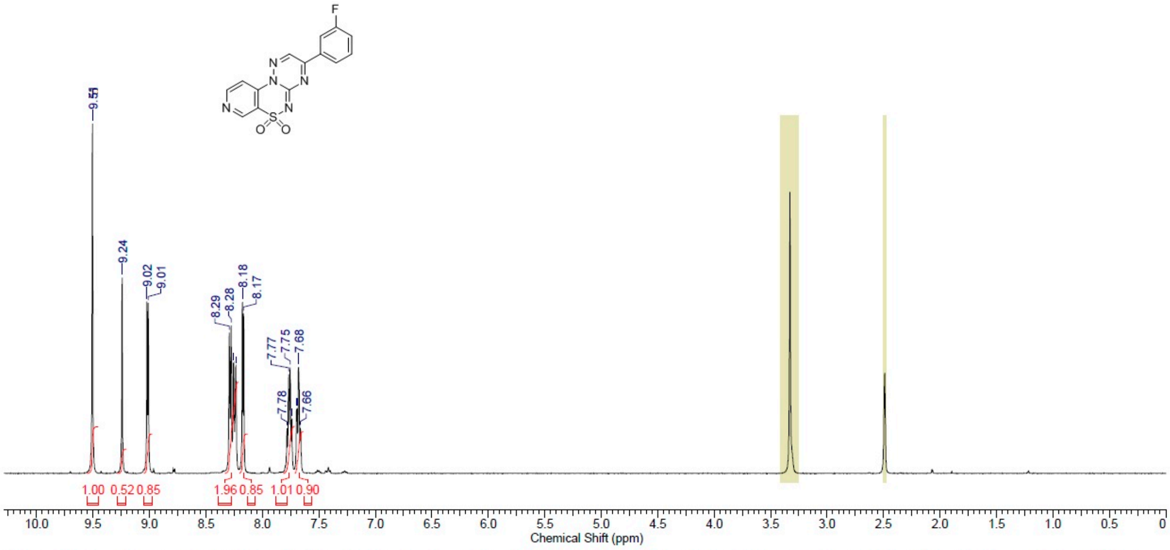

No.	(ppm)	(Hz)	Height	No.	(ppm)	(Hz)	Height	No.	(ppm)	(Hz)	Height	No.	(ppm)	(Hz)	Height	No.	(ppm)	(Hz)	Height
1	7.66	3830.5	1.5578	5	7.74	3868.1	1.8784	9	8.17	4081.5	5.4901	13	8.28	4137.2	5.0772	17	9.24	4619.6	6.7240
2	7.68	3838.8	3.6495	6	7.75	3875.9	3.6301	10	8.18	4087.4	5.8796	14	8.29	4145.0	4.8343	18	9.51	4751.0	12.0000
3	7.69	3845.2	2.2345	7	7.77	3882.3	3.3977	11	8.24	4116.2	3.6717	15	9.01	4503.4	5.8423				
4	7.70	3847.1	2.2145	8	7.78	3890.1	1.5506	12	8.25	4125.9	3.7976	16	9.02	4509.3	5.8947				

Figure S16. ^{1}H-NMR (500 MHz, DMSO-d_6) spectrum of compound **22.**

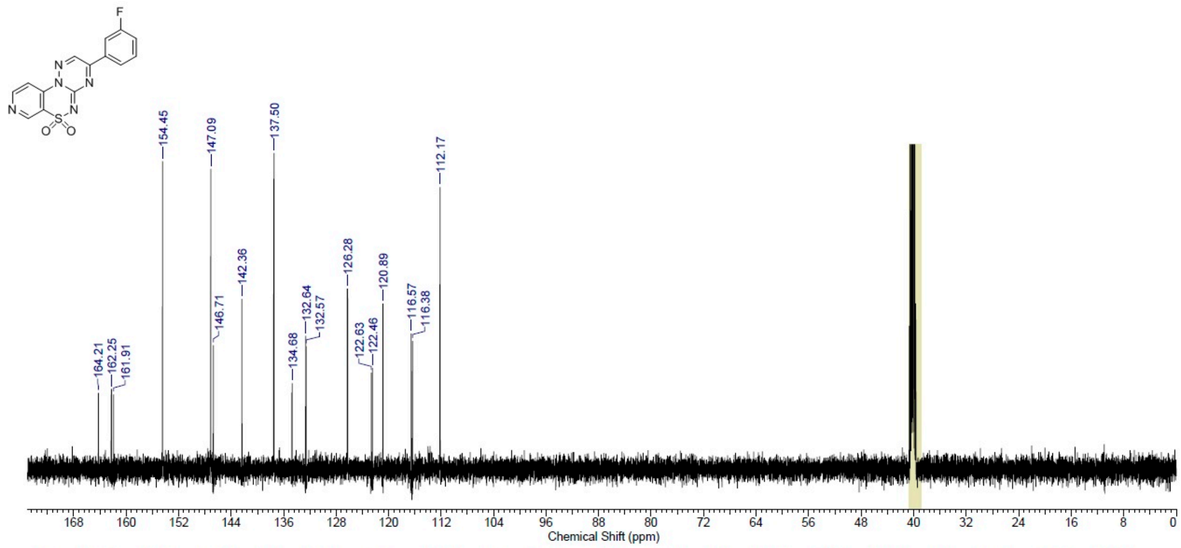

No.	(ppm)	(Hz)	Height	No.	(ppm)	(Hz)	Height	No.	(ppm)	(Hz)	Height	No.	(ppm)	(Hz)	Height	No.	(ppm)	(Hz)	Height
1	112.17	14098.5	10.7094	5	122.46	15392.6	3.8289	9	132.57	16662.9	4.6603	13	137.50	17282.1	12.0000	17	154.45	19412.6	11.6885
2	116.38	14628.1	4.8690	6	122.63	15413.7	3.6543	10	132.64	16671.3	5.0573	14	142.36	17893.3	6.4651	18	161.91	20351.0	2.8452
3	116.57	14651.4	5.1495	7	126.26	15869.5	6.2918	11	134.68	16927.7	3.2686	15	146.71	18440.4	4.7057	19	162.25	20394.0	3.0493
4	120.89	15195.4	6.2843	8	126.28	15872.1	6.8625	12	134.74	16935.6	2.9063	16	147.09	18488.3	11.4080	20	164.21	20639.5	2.8884

Figure S17. ^{13}C-NMR (125 MHz, DMSO-d_6) spectrum of compound **22**.

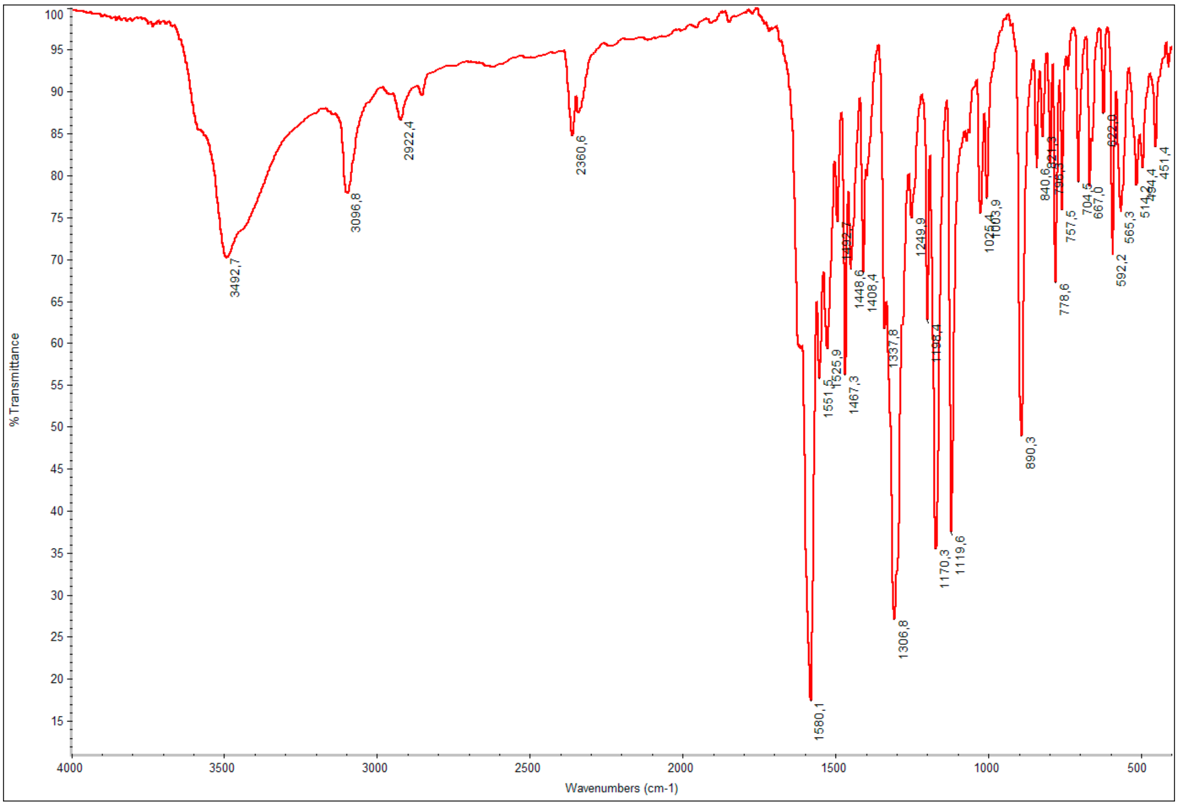

Figure S18. IR spectrum of compound **22**.

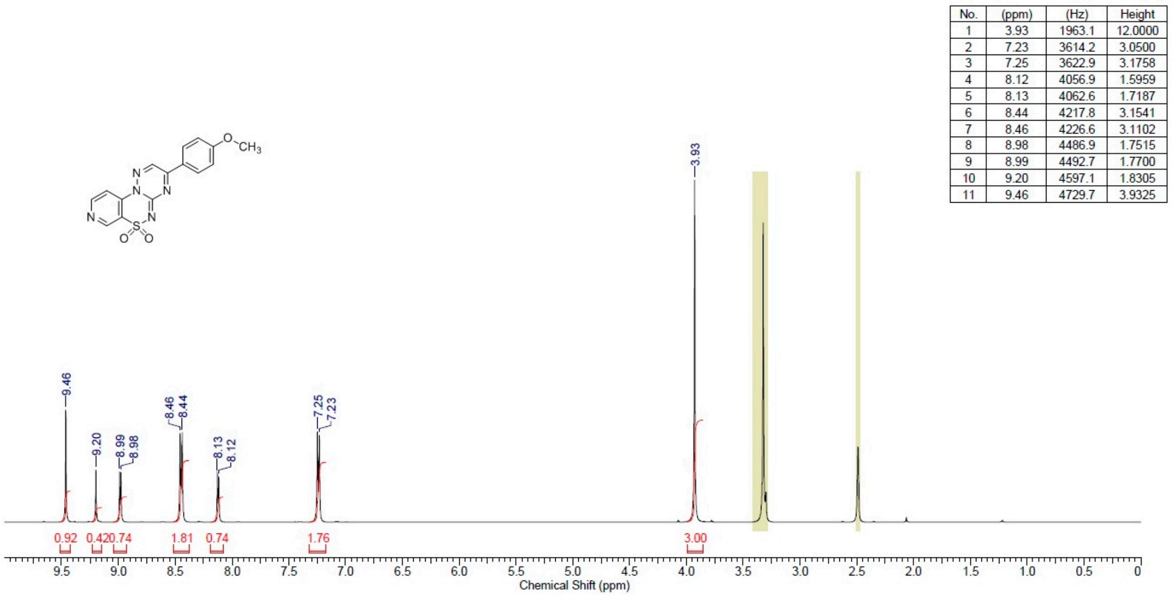

No.	(ppm)	(Hz)	Height
1	3.93	1963.1	12.0000
2	7.23	3614.2	3.0500
3	7.25	3622.9	3.1758
4	8.12	4056.9	1.5959
5	8.13	4062.6	1.7187
6	8.44	4217.8	3.1541
7	8.46	4226.6	3.1102
8	8.98	4486.9	1.7515
9	8.99	4492.7	1.7700
10	9.20	4597.1	1.8305
11	9.46	4729.7	3.9325

Figure S19. ^{1}H-NMR (500 MHz, DMSO-d_6) spectrum of compound **26**.

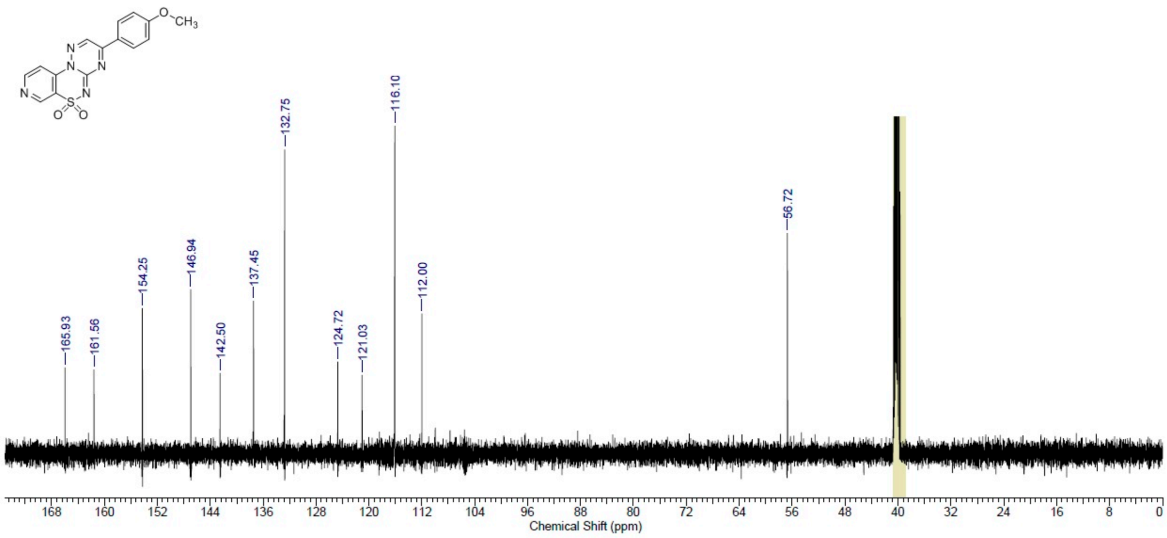

No.	(ppm)	(Hz)	Height	No.	(ppm)	(Hz)	Height	No.	(ppm)	(Hz)	Height	No.	(ppm)	(Hz)	Height
1	56.72	7129.0	8.0796	4	121.03	15212.6	2.8747	7	137.45	17276.4	5.5841	10	154.25	19388.0	5.3277
2	112.00	14077.5	5.1208	5	124.72	15675.8	3.3647	8	142.50	17911.3	2.9391	11	161.56	20306.7	3.0833
3	116.10	14593.0	12.0000	6	132.75	16685.3	11.1259	9	146.94	18468.5	6.0173	12	165.93	20856.4	3.1557

Figure S20. ^{13}C-NMR (125 MHz, DMSO-d_6) spectrum of compound **26**.

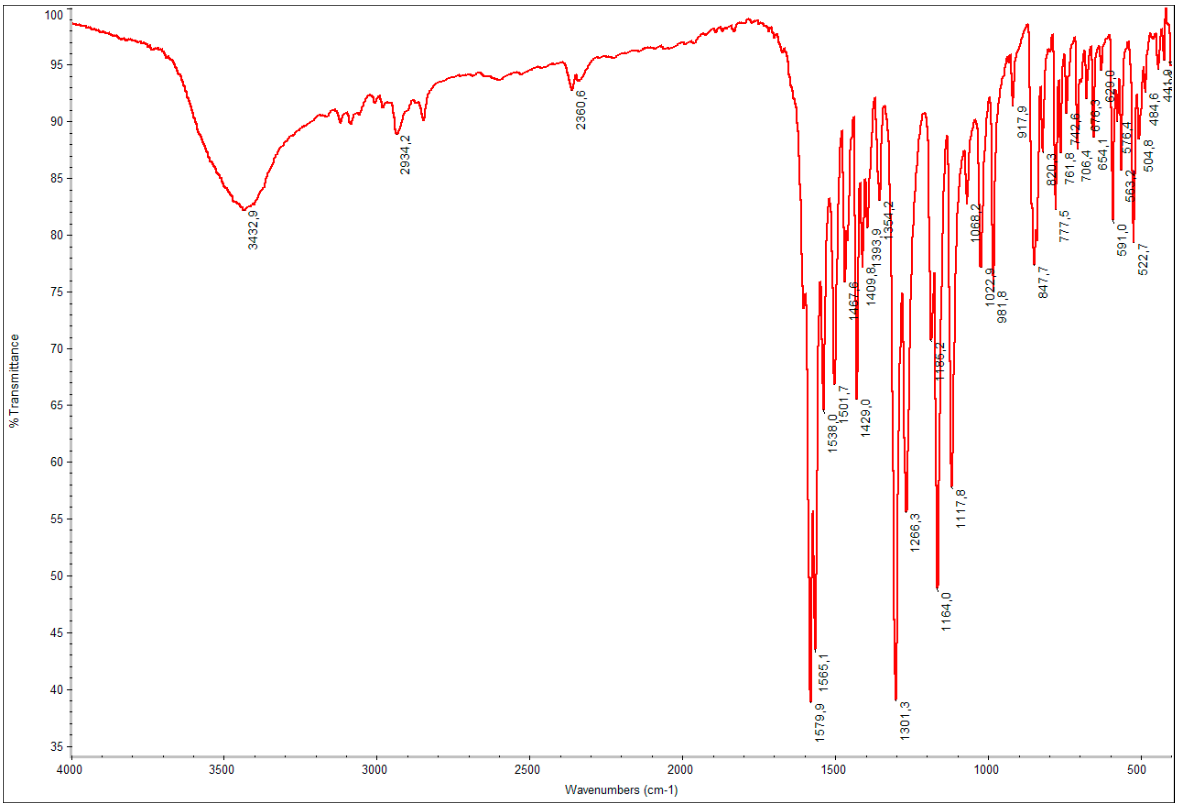

Figure S21. IR spectrum of compound **26**.

2D-NMR Spectra of Compound 7

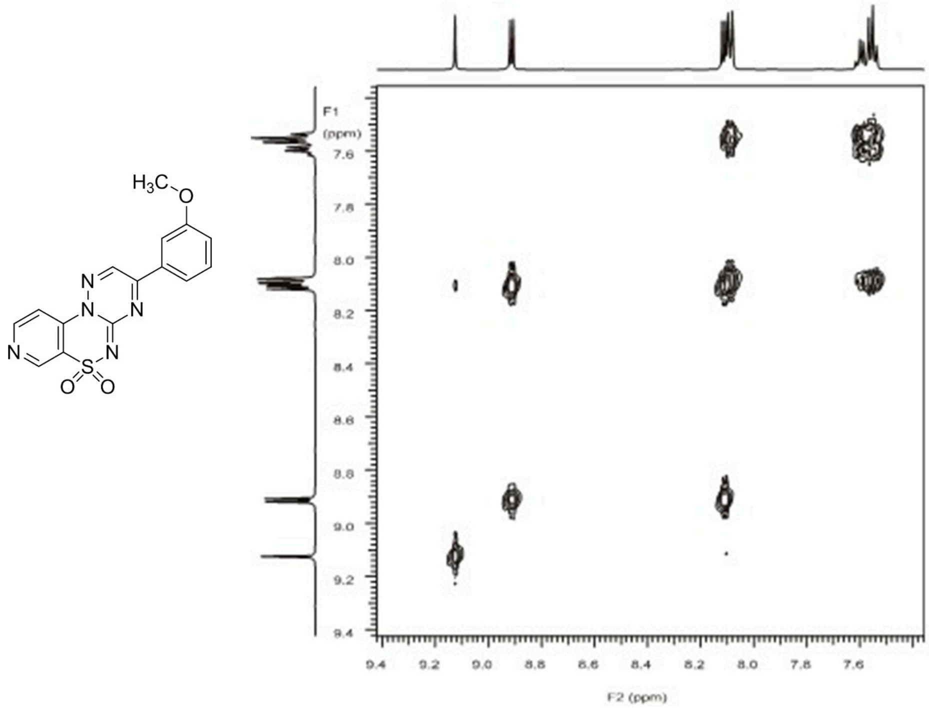

Figure S22. COSY spectrum of compound **7**.

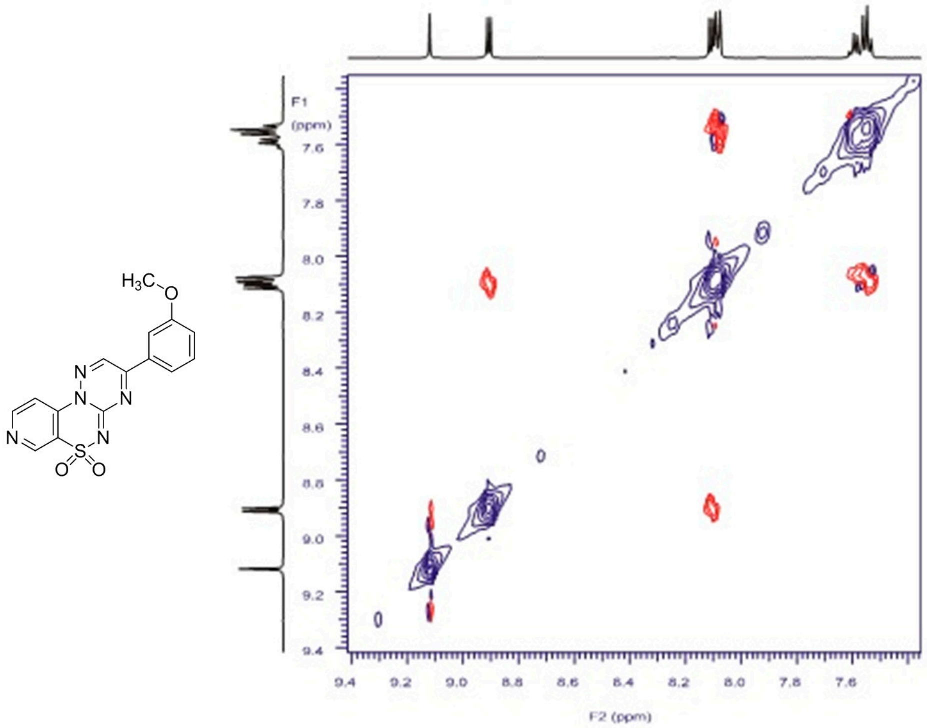

Figure S23. ROESY spectrum of compound **7**.

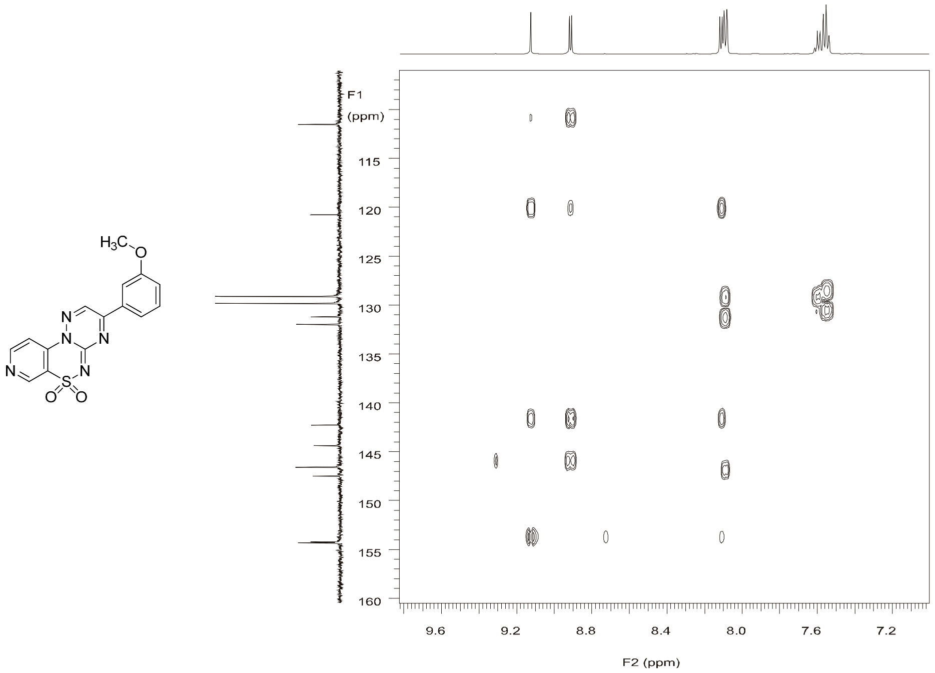

Figure S24. HMBC spectrum of compound **7**.

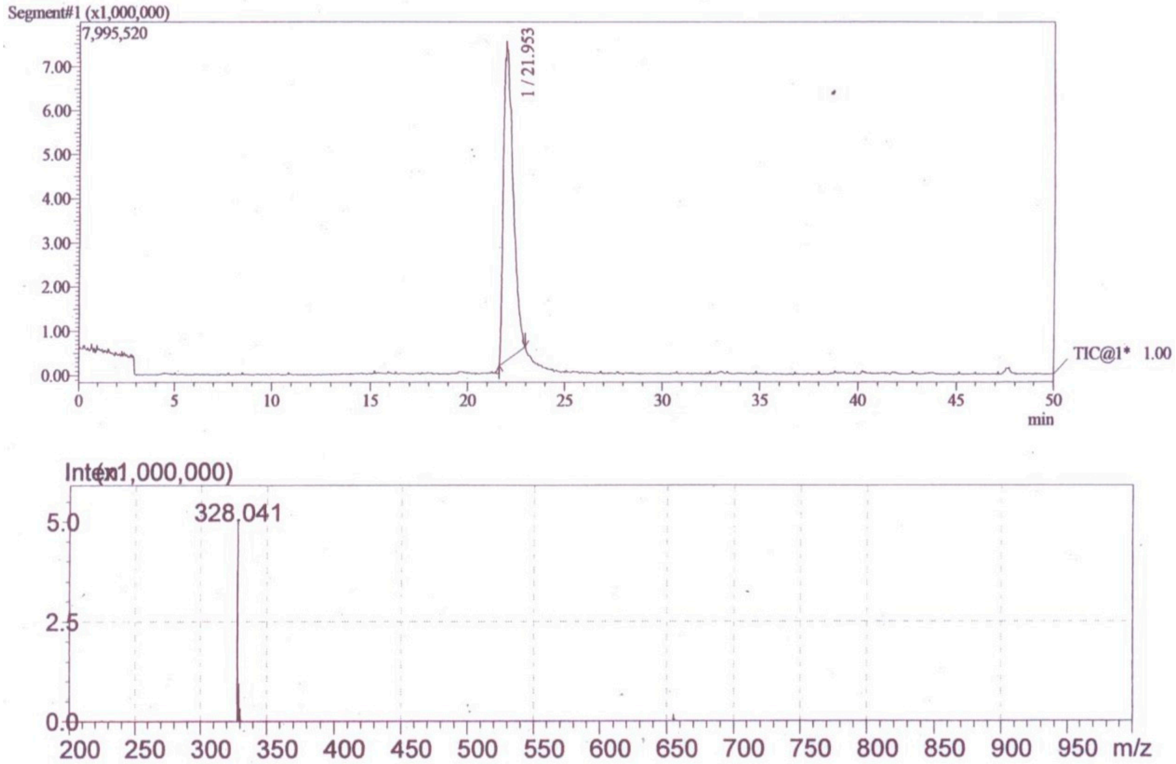

Figure S25. LC-MS spectrum of compound **7**.

2D-NMR Spectra of Compound 25

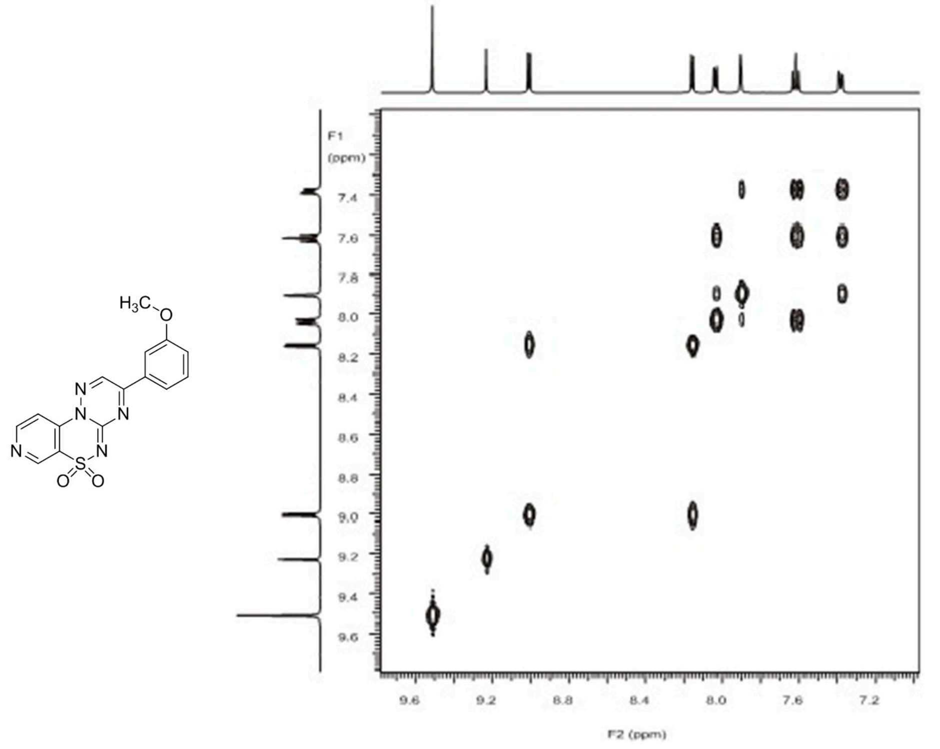

Figure S26. COSY spectrum of compound **25**.

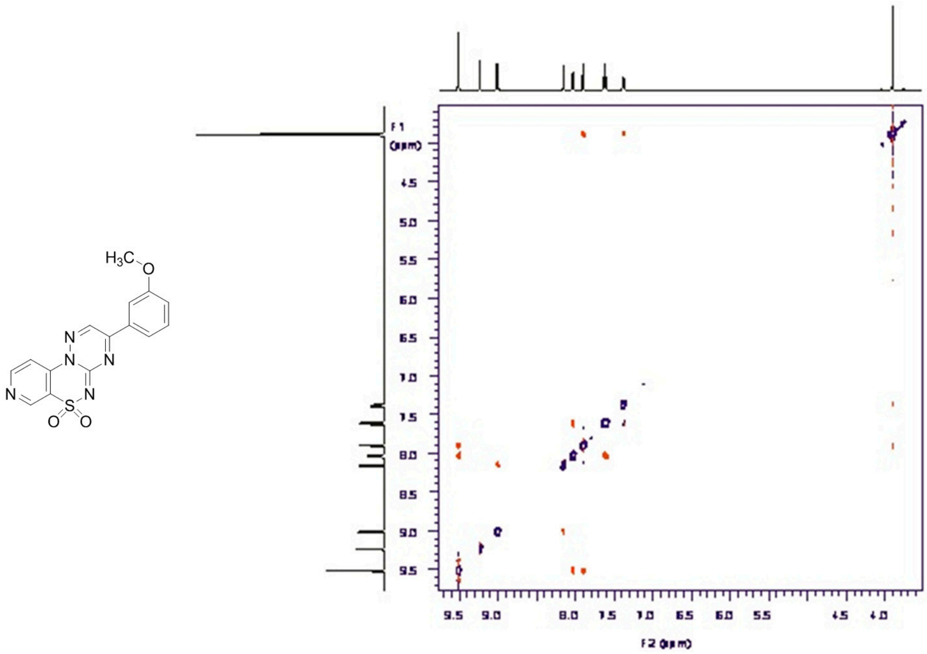

Figure S27. ROESY spectrum of compound **25**.

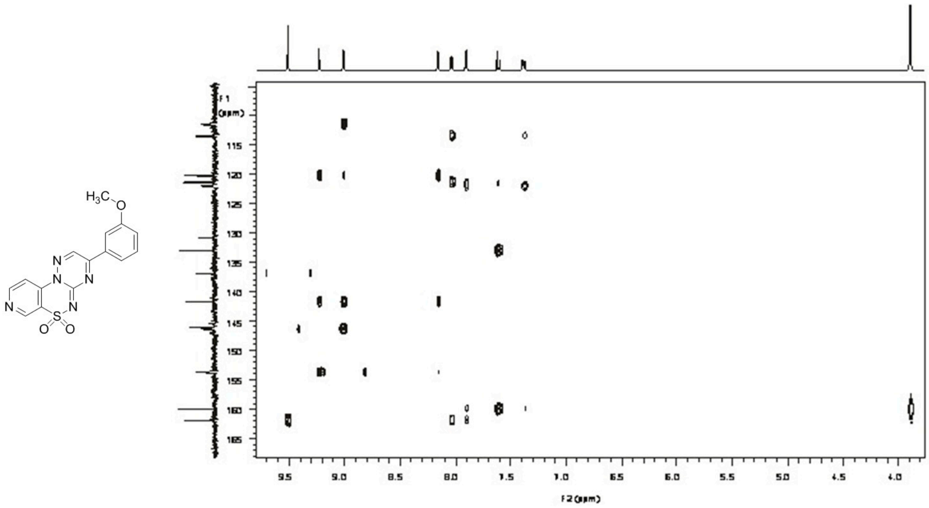

Figure S28. HMBC spectrum of compound **25**.
